# Direct visualization of the transition status during neural differentiation by dual-fluorescent reporter human pluripotent stem cells

**DOI:** 10.1016/j.stemcr.2022.07.001

**Published:** 2022-08-04

**Authors:** Gwanghyun Park, Minkyung Shin, Wonyoung Lee, Akitsu Hotta, Taeko Kobayashi, Yoichi Kosodo

**Affiliations:** 1 Korea Brain Research Institute (KBRI), Daegu 41068, Republic of Korea; 2 Center for iPS Cell Research and Application (CiRA), Kyoto University, Kyoto 606-8507, Japan; 3 Research Center for Dynamic Living Systems, Graduate School of Biostudies, Kyoto University, Kyoto 606-8501, Japan

**Keywords:** human iPSCs, CRISPR-Cas9, genome edition, NEUROG2, TUBB3, REELIN, brain organoids, transplantation, live imaging

## Abstract

Human induced pluripotent stem cells (hiPSCs) can differentiate into neurons and glia via neural progenitor cells and are widely used for neurogenic studies. However, directly visualizing the transition status during the neural differentiation of live cells is difficult. Here, targeting NEUROG2 (NGN2) and TUBB3 as markers of neurogenic cells and neurons, respectively, we established TUBB3^EGFP^/NGN2^TagRFP^ dual-reporter hiPSCs using CRISPR-Cas9 technology. We induced the differentiation of cortical neurons from dual-reporter hiPSCs, successfully visualizing cell-fate conversion in two-dimensional (2D) culture and 3D organoids. The reporter cells were used to monitor drug effects to enhance neural induction, responses to gene knockdown, transplantation to the embryonic mouse brain, and live imaging at single-cell resolution. Notably, the earliest REELIN-positive neurons showed a distinctive migration pattern, and their production was accelerated by HES1-function loss. Together, these results demonstrate the potential for dual-reporter hiPSCs in therapeutic neural regeneration strategies and studies on human cortical development.

## Highlights

• Two-color fluorescent reporter hiPSC lines are established by genome editing
• Neural differentiation is characterized by 2D and 3D culture methods
• The dual-reporter line is applied to evaluate drug and gene functions
• HES1 knockdown accelerates the production of the earliest REELIN-positive neurons

## Graphical abstract

**Figure:**
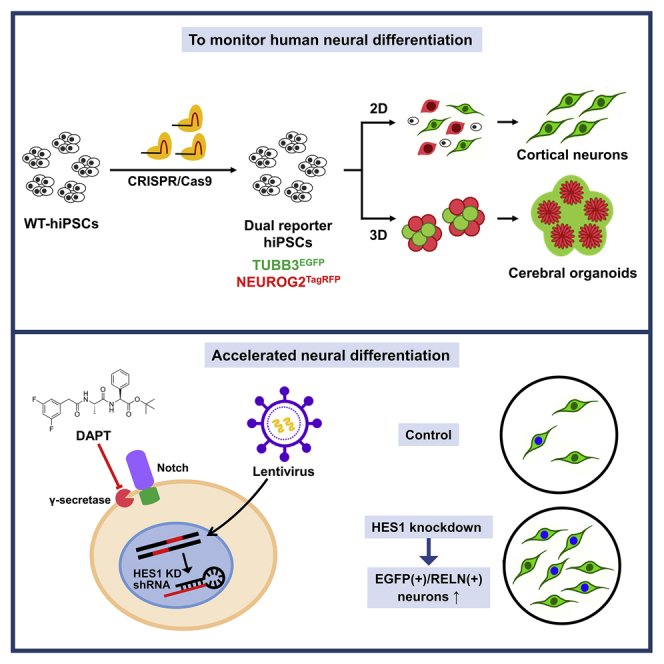

## Teaser

Park et al. have established and demonstrated the successful use of TUJ1^EGFP^/NGN2^TagRFP^ dual-reporter induced pluripotent stem cells to monitor human neural differentiation in 2D and 3D culture systems. They also document the responses of fluorescent signals to drugs and genetic manipulations affecting neural differentiation. Furthermore, the knock down of HES1 accelerates the production of the earliest REELIN-positive neurons.

## Introduction

Human induced pluripotent stem cells (hiPSCs) have many advantages for modeling development and disease, cell therapy and drug screening. Numerous studies have reported the production of neural and glial cells in the central nervous system (CNS) via neural progenitor cells from hiPSCs and human embryonic stem cells (ESCs). Considering the use of such cells for therapeutic purposes such as drug testing and transplantation in animal models, monitoring the differentiation status of living cells would provide vital information. To do this, the introduction of fluorescent reporter protein coding sequences into the loci of specific marker genes by genome-editing technology has shown promise (Hotta and Yamanaka, 2015).

To date, several fluorescent reporter cell lines that distinguish the neural differentiation status have been established, such as NEUROG2 (NGN2) for neurogenic cells (Li et al., 2015) and tyrosine hydroxylase for dopaminergic neurons (Calatayud et al., 2019). Although these “single” fluorescent reporter cells can distinguish differentiated cells from pluripotent stem cells, differentiation into somatic cells generally requires many steps, and several intermediate cell populations appear prior to terminal differentiation. To trace multiple differentiation statuses in live cells, a dual-fluorescent reporter system has been shown to be useful for some cellular lineages. Various cardiac cells derived from TBX5^Clover2^/NKX2-5^TagRFP^ hiPSCs were used for precise drug testing (Zhang et al., 2019). Likewise, PAX7^tdTomato^/MYF5^EGFP^ and NKX2-1^EGFP^/TP63^tdTomato^ reporter cells allowed the successful characterization of skeletal muscle progenitors (Wu et al., 2018) and airway basal stem cells (Hawkins et al., 2021), respectively.

Regarding the cortical neural lineage, neuroepithelial stem cells typically differentiate into radial glial cells and then into intermediate progenitors. Such cells are categorized as neural progenitors because of their ability to produce postmitotic neurons. Intriguingly, a specific subtype of neural progenitors named “outer glial cells” are abundant in mammals within the gyrified cortex and may play critical roles in neocortical expansion (Kalebic and Huttner, 2020). Therefore, monitoring transient status during neural differentiation with multiple reporters would become a strong and versatile approach not only for therapeutic strategies, such as screening and efficient production of specific neurons, but also for elucidating developmental programs, especially in humans.

In this study, using NGN2 and Tubulin β-III (TUBB3) as markers of neurogenic cells and neurons, respectively, we established TUBB3^EGFP^/NGN2^TagRFP^ dual-color fluorescent reporter hiPSCs followed by characterization using two-dimensional (2D) and 3D culture systems. The dual-reporter cells were used to monitor drug responses, gene knockdown (KD), and transplantation to embryonic mouse brains. We further performed live imaging to assess migration characteristics. By combining these approaches, we discovered that the appearance of the earliest neurons in the human neural lineage was accelerated by the KD of HES1, a basic helix-loop-helix transcription factor. Furthermore, the earliest induced neurons were enriched for the REELIN (RELN)-positive neural subtype. These results demonstrate the usefulness of dual-color reporters to visualize shifts in human neural lineage in live cells for a number of applications.

## Results and discussion

### Establishment and characterization of TUBB3^EGFP^/NGN2^TagRFP^ dual-reporter hiPSCs

**Figure 1:**
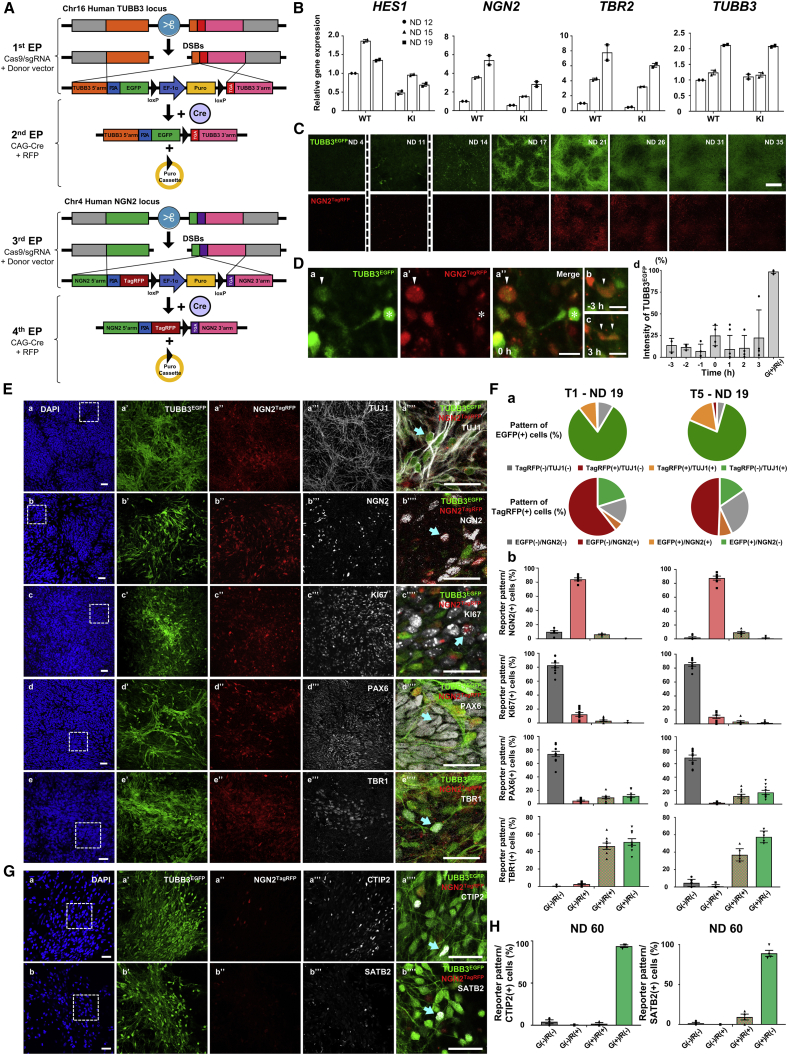
Establishment and characterization of dual-reporter hiPSC-derived neural cells (A) Schematic of EP to establish dual-reporter hiPSC lines. (B) qRT-PCR analysis of the original (wild-type [WT]) and dual-reporter hiPSC (T1)-derived neural cells (KI) on NDs 12, 15, and 19. The expression level for each gene was normalized to that on ND 12 from WT. Independent experiments (IEs), 2. (C) Sequential images during neural differentiation (T1, up to ND 35) at the same position on the grid culture dish. Note that images at unspecified positions are presented for NDs 4 and 11 because of faint signals at the stages. Bar: 300 μm. (D) (a–c) Division of R^+^ cell (arrowheads) (NDs 14–15, T1). Time point 0 corresponds to the time just before the separation of daughter cells. Bars: 15 μm. (d) EGFP signal intensity of 4 dividing cells (5 IEs). A G^+^/R^−^ cell (asterisks) was used as the reference (100%) among recorded images. (E) Immunostaining images obtained using TUJ1 (a), NGN2 (b), KI67 (c), PAX6 (d), and TBR1 (e) antibodies fixed on ND 19. (a’’’’)–(e’’’’) show magnified views of the dashed squares in (a)–(e), respectively. The arrows indicate TUJ1^+^/G^+^ (a’’’’), NGN2^+^/R^+^ (b’’’’), KI67^+^/R^+^ (c’’’’), PAX6^+^ and G^−^/R^−^ (d’’’’), and TBR1^+^/G^+^ cells (e’’’’). Bar: 30 μm. (F) (a) (Top) Populations of R^+^ and TUJ1^+^ among G^+^ cells. (Bottom) Populations of G^+^ and NGN2^+^ among R^+^ cells. (b) Quantification of G^+^ and R^+^ among the NGN2^+^, PAX6^+^, TBR1^+^, and KI67^+^ cells in (E). Number of fields, 6–10 for each T1 and T5 from 3 IEs. (G) Immunostaining images obtained using CTIP2 (a) and SATB2 (b) antibodies after long-term neural differentiation (T5, ND 60). (a’’’’) and (b’’’’) show magnified views of the dashed squares in (a) and (b), respectively. The arrows indicate CTIP2^+^/G^+^ (a’’’’) and SATB2^+^/G^+^ cells (b’’’’). Bar: 30 μm. (H) Quantification of G^+^ and R^+^ among the CTIP2^+^ and SATB2^+^ cells in (G). Number of fields, 3–4 from 2 IEs.

To generate dual-reporter hiPSCs, multi-step electroporation (EP) of CRISPR-Cas9 genome editing and Cre-recombination were performed (Figure 1A). Single-guide RNAs (sgRNAs) and homology arms were designed to add the coding sequences of EGFP and TagRFP with the self-cleaving peptide P2A (Kim et al., 2011) near the stop codons of TUBB3 and NGN2, respectively (Figures 1A and S1). For the host hiPSCs, the RNA-based reprogrammed RPChiPS771 line, which has sufficient ability to differentiate into three germ layers and produce cortical neurons (Iwashita et al., 2019), was selected among 4 hiPSC lines. We selected TUBB3^EGFP^/NGN2^TagRFP^ heterozygous knockin (KI) clones (Figure S1) after confirming their normal karyotype and pluripotency (Figure S1) in lines with different sgRNA target sites for NGN2 (named T1 and T5). Subsequently, we induced the differentiation of neural cells into cortical cells using the 2D culture method (Espuny-Camacho et al., 2013) and assessed whether the genome modifications influenced neural differentiation by examining the transcript levels of marker genes (*HES1*, *NGN2*, *TBR2*, and *TUBB3*). The neural induction profiles of cells derived from the dual-reporter and parental hiPSCs showed similar fluctuation patterns along 12, 15, and 19 days (Figure 1B). We then monitored the expression of fluorescent proteins during neural differentiation for 5 weeks (Figures 1C and S2). In both the T1 and T5 lines, distinct expression patterns of EGFP and TagRFP were observed as early as neural differentiation day (ND) 11, whereas these signals were not visible in the initial stage (ND 4). Notably, the EGFP signal increased and was maintained over time, while the TagRFP signal peaked at approximately ND 20–24 and thereafter became weaker (Figures 1C and S2). Time-lapse recordings allowed us to identify dividing NGN2^TagRFP^-positive (R^+^) cells (Figure 1D; Video S1). Although a faint signal of EGFP was detected during mitosis, the intensity level was far less than TUBB3^EGFP^-positive (G^+^) cells without a TagRFP signal. These results indicated that R^+^ cells appeared as neurogenic cells and then were consumed to produce neurons, while G^+^ cells remained as postmitotic neurons.

Video S1. Time-lapse movie file for Figure 1D (12 min/frame)

To perform further analyses at the cellular resolution, immunostaining was used to evaluate neural cells at ND 19, the time point at which proliferative cells, progenitor cells, and postmitotic neurons exist simultaneously (Figures 1E and 1F). We first compared endogenous TUBB3 and NGN2 proteins with EGFP and TagRFP fluorescent reporters, respectively. The vast majority of G^+^ cells overlapped with the antibody for TUBB3 (TUJ1) staining (91% [T1] and 94% [T5]). In the case of R^+^ cells, overlapped cells with the antibody for NGN2 were 65% (T1) and 57% (T5), and 20% (T1) and 15% (T5) were G^+^/NGN2^−^. Among NGN2^+^ cells, 84% (T1) and 88% (T5) were R^+^. A part of KI67-, a proliferative marker, positive cells were R^+^ (12% [T1] and 10% [T5]). TBR1, a marker of newborn cortical neurons, overlapped not only with the G^+^ populations but also with G^+^/R^+^ cells (46% [T1] and 37% [T5]). These results suggest that TagRFP protein can remain temporally after endogenous NGN2 protein is diminished in newborn neurons. In future studies, use of destabilized RFP variants might improve the temporal sensitivity. We further assessed long-term neural differentiation (ND 60). Among CTIP2^+^ (marker of layer 5 neurons) and SATB2^+^ (marker of layer 2/3 neurons) cells, 94% and 89% were G^+^, respectively, indicating that EGFP continued to be expressed in terminally differentiated neurons (Figures 1G and 1H). Taken together, TUBB3^EGFP^/NGN2^TagRFP^ dual-reporter hiPSCs can differentiate into cortical neurons, and the expression of each fluorescent reporter reflects neural fate conversion.

### Cerebral organoid generation and xenograft transplantation using dual-reporter cells

**Figure 2:**
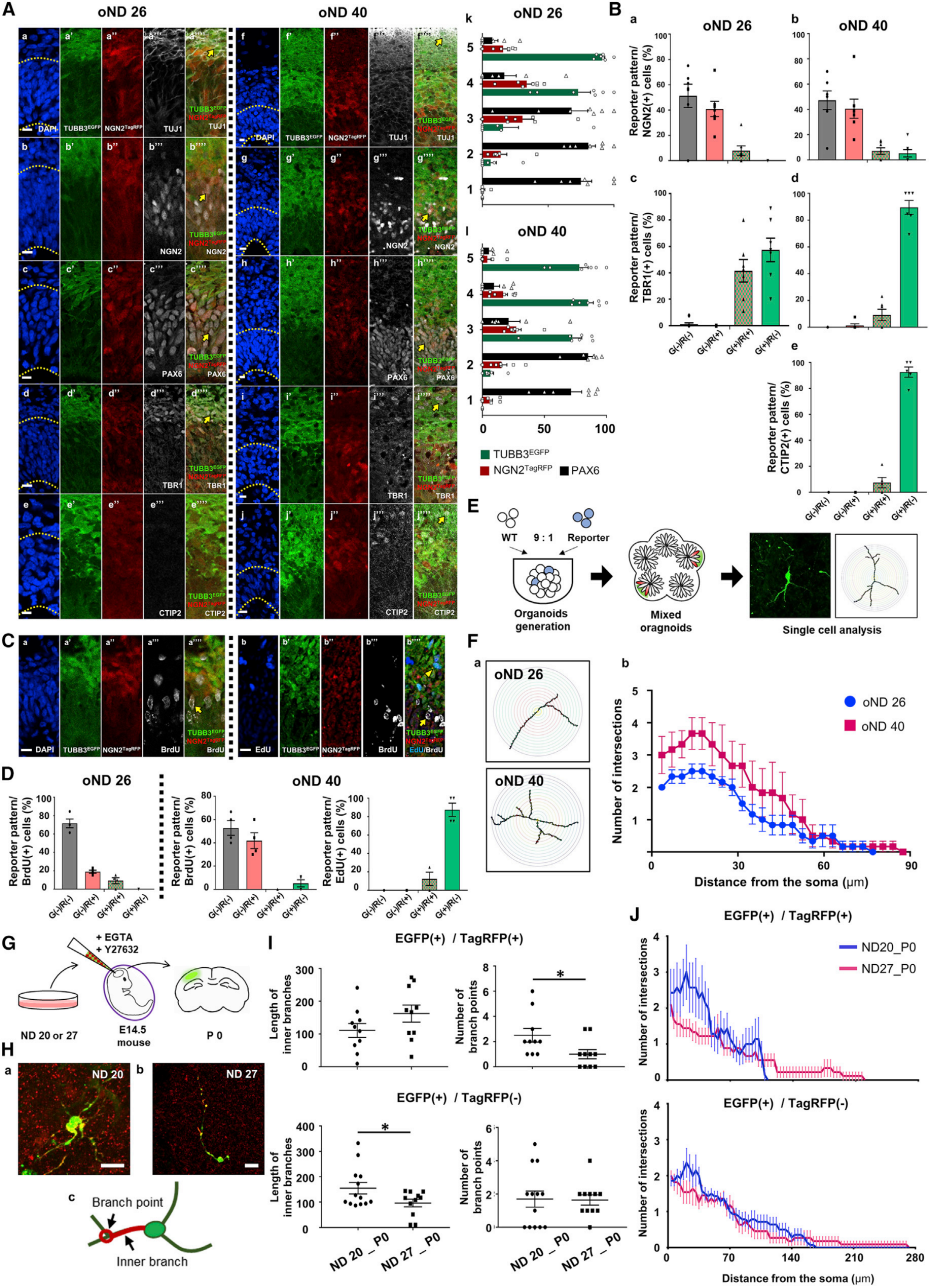
Analysis of dual-reporter hiPSC-derived neural cells in brain organoids and the developing mouse brain (A) Immunostaining images obtained using TUJ1 (a and f), NGN2 (b and g), PAX6 (c and h), TBR1 (d and i), and CTIP2 (e and j) antibodies fixed on oNDs 26 and 40 (T1). The arrows indicate TUJ1^+^/G^+^ (a’’’’ and f’’’’), NGN2^+^/R^+^ (b’’’’ and g’’’’), PAX6^+^ and G^−^/R^−^ (c’’’’ and h’’’’), TBR1^+^/G^+^ (d’’’’ and i’’’’), and CTIP2^+^/G^+^ (j’’’’) cells. Dotted line (a–j): border of VZ. Bar: 10 μm. (k and l) Ratios of G^+^, R^+^, and PAX6^+^ cells at oNDs 26 (c) and 40 (h) (%, per DAPI). The cortical structure from apical to basal surface is evenly divided into bins 1–5. Number of fields, 7 from 7 organoids (3 IEs). (B) Quantification of G^+^ and R^+^ among the NGN2^+^, TBR1^+^, and CTIP2^+^ cells in (A). Number of fields, 7 from 7 organoids (3 IEs). (C) BrdU/EdU labeling to assess the proliferation and migration (T5). (a) BrdU was applied 4 h before fixation on oND 26. (b) EdU and BrdU were applied for 14 days and 4 h, respectively, before fixation on oND 40. The arrows and arrowheads indicate BrdU^+^/R^+^ (a’’’’ and b’’’’) and EdU^+^/G^+^ (b’’’’) cells, respectively. Bar: 10 μm. (D) Quantification of the images in (C). Number of fields, 4 from 4 organoids (2 IEs). (E) Schematic of mixed organoid generation using WT and dual-reporter hiPSCs. (F) Representative tracing images of G^+^ cells as analyzed by the Sholl method in mixed organoids (a) and quantification (b) (6 T1 cells at both oNDs 26 and 40 from 2 IEs). (G) Schematic of the transplantation of dual-reporter cells into the lateral ventricles of embryonic mice. (H) Representative images of G^+^/R^+^ cells using ND 20 (a) and 27 (b) donor cells (T1). (c) Parameters for morphological quantification. Bar: 20 μm. (I) Quantification of the parameters indicated in (H) for G^+^/R^+^ (n = 10 for NDs 20 and 27) and G^+^ cells (n = 13 and 11 for NDs 20 and 27, respectively) (3 IEs). (J) Quantification of the number of intersections of grafted dual-reporter cells (same sample as I).

We attempted to characterize the dual-reporter cells in a 3D environment in applications involving cerebral organoids and xenograft transplantation to the developing mouse brain. We cultured organoids for 26 and 40 days (oNDs 26 and 40) after the initiation of embryoid body (EB) formation and then evaluated the expression of fluorescent reporters and cell positions both inside and outside the ventricular zone (VZ), as defined by PAX6, a neural stem cell (NSC) marker, immunostaining (Figures 2A and S3). G^+^ cells were localized peripherally in the VZ, while R^+^ cells were observed between the G^+^ layer and the VZ. Furthermore, at oND 40, the VZ was thinner, while the TUJ1 immunopositive G^+^-layer was thicker than that on oND 26 (Figure 2A). These results indicated that TagRFP and EGFP were sequentially expressed while cells were migrating out from the VZ. Among NGN2^+^ cells at both oNDs 26 and 40, approximately 40% and 7%–8% were R^+^ and G^+^/R^+^, respectively, while 50% of cells were R^−^, mostly localizing at the apical region of the VZ (Figures 2A and 2B). This staining feature resembled the pattern of the Neurogenin2 fluorescent reporter in the developing cortices of *Ngn2*-EGFP Tg mice (Figure S4). We further evaluated the expression of TBR1 and CTIP2 to examine neural subtypes in the organoids (Figures 2A and 2B). Around half of TBR1^+^ cells were G^+^/R^+^ at oND 26, while most of TBR1^+^ cells were G^+^/R^−^ at oND 40. The results suggest that the generation of TBR1^+^ neurons is intensive at oND 26 and decreases at oND 40. This is because the TagRFP remaining in G^+^ cells should represent newly born neurons, as observed in 2D culture. CTIP2^+^ cells were rarely observed at oND 26 while appearing at oND 40, which reflects the chronologicity of corticogenesis.

We next analyzed the mitotic activity of R^+^ cells and the migration of G^+^ cells in the organoids using EdU and BrdU, thymidine analogs incorporated by proliferative cells during the S phase. First, we treated oND 26 cells with BrdU prior to fixation and observed that BrdU^+^ cells were sparsely distributed in the VZ (Figure 2C). Importantly, 19% of BrdU^+^ cells were R^+^, indicating that they expressed NGN2^TagRFP^ during or immediately after the S phase. Next, EdU was supplied for 4 h on oND 26, and BrdU was administered on oND 40. Most EdU^+^ cells were G^+^ (88%), while 42% of BrdU^+^ cells were R^+^ (Figure 2D). These results confirm that R^+^ cells have the ability of mitosis in the neurogenic niche in organoids as well as in 2D culture, while G^+^ cells migrate to the basal side as postmitotic neurons.

We performed quantitative morphological assays at the single-cell level by culturing heterogeneous cell populations to form “mosaic” organoids (Figure 2E). Dual-reporter and parental hiPSCs were mixed at a ratio of 1 to 9 at the EB-formation step. Sholl analysis revealed that G^+^ cells had fewer intersections close to the soma at oND 26 compared with at oND 40 (Figure 2F), indicating an increase of neurites in organoids over time.

The morphology of dual-reporter cells at the single-cell level was further examined in the developing mouse cortex using an efficient transplantation method that we previously established (Nagashima et al., 2014) (Figure 2G). Dual-reporter cells were transplanted into the embryonic day 14.5 (E14.5) brain on NDs 20 and 27, followed by dissection at birth. Due to the small number of R^+^ cells, we compared the morphological parameters of the G^+^/R^+^ and G^+^ populations (Figure 2H). More branch points and intersections were observed in the G^+^/R^+^ population transplanted on ND 20 than on those transplanted on ND 27, a feature not observed in the G^+^ population (Figures 2I and 2J). These results indicated that the number of branched shapes in newborn neurons were directly associated with the period of neural induction, with more branched shapes being correlated with a shorter period of induction. Accordingly, dual-reporter cells allow complex morphological analyses of specific cell types after xenograft transplantation.

### Application of dual-reporter cells for drug response and gene function analyses

**Figure 3:**
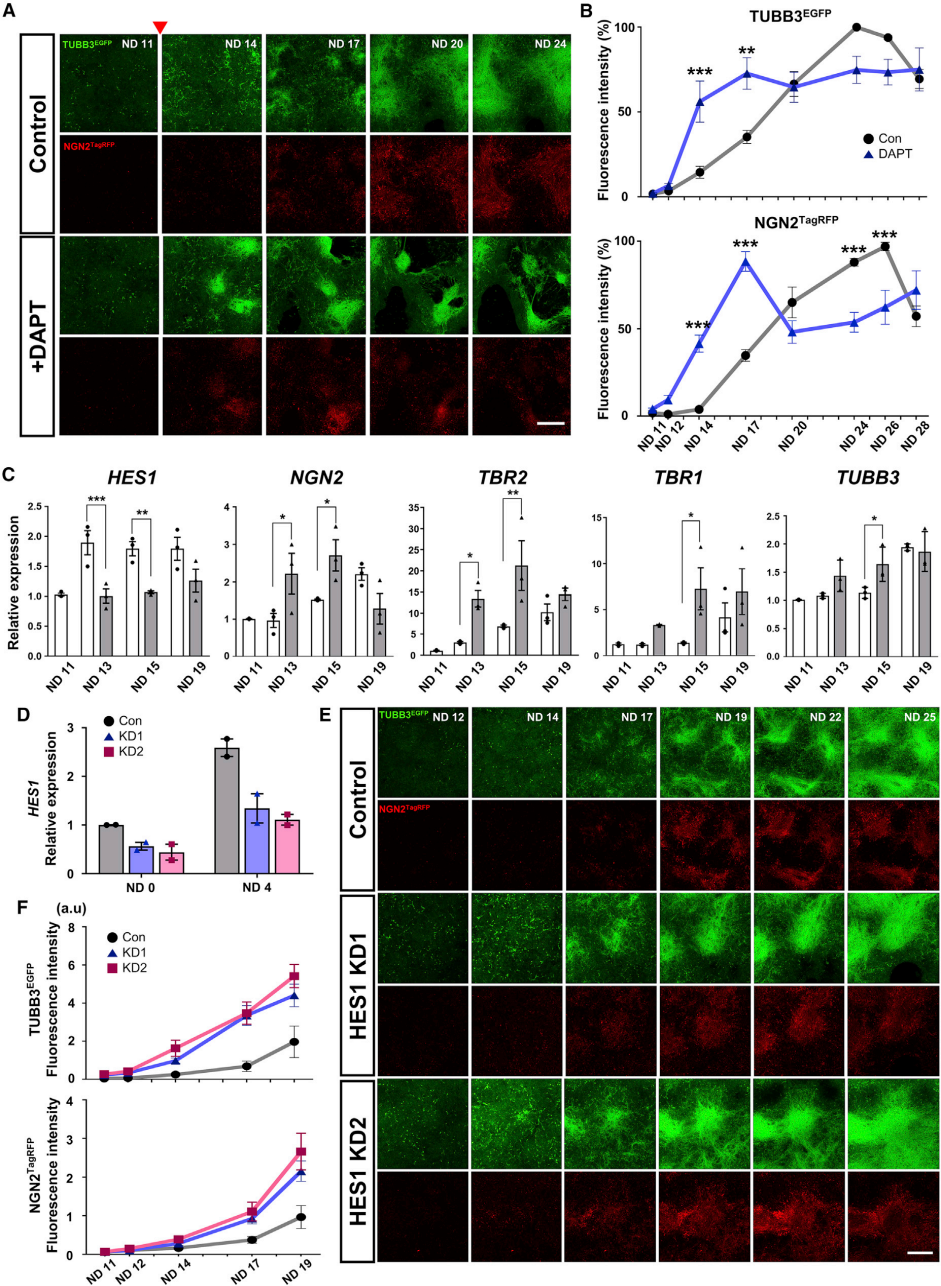
Monitoring the temporal potentiation of neural differentiation using drug treatment and gene KD strategies (A) Sequential images of G^+^ and R^+^ cells (T1) at the same position on the culture dish after treatment with DAPT (5 μM) starting on ND 11 (arrowhead). Bar: 300 μm. (B) Quantification of the fluorescence intensities of the images in (A). Gray for the control and blue for the DAPT-treated cells. Number of fields, 9–10 from 3 IEs. (C) qRT-PCR analysis of neural marker genes in the control (white) and DAPT (gray)-treated cells. The expression levels were normalized by those in the control group on ND 11 (3 IEs). (D) qRT-PCR analysis of HES1 KD dual-reporter cells (T1) on NDs 0 (iPSC stage) and 4. The expression levels were normalized to those in the control group on ND 0. Two different shRNA constructs (KD1 and 2) were utilized (2 IEs). (E) Sequential images of G^+^ and R^+^ cells (T1) with HES1 KD at the same position on the culture dish. Bar: 300 μm. (F) Quantification of the fluorescence intensities (arbitrary unit) of the images in (E). Number of fields, 7 from 2 IEs.

Reporter cells can be used to monitor the differentiation status of cells in real time during culture. We next assessed the applicability of dual-reporter cells for this purpose by evaluating the Notch signaling pathway, a key regulator of NSC proliferation and differentiation (Kobayashi and Kageyama, 2011). We used dual-reporter cells to visualize neural lineage conversion via the Notch signal inhibitor DAPT, a gamma-secretase inhibitor (Crawford and Roelink, 2007). DAPT was administered on ND 11, and the expression of fluorescent reporters was monitored by sequential imaging. On NDs 14–17, the numbers of both G^+^ and R^+^ cells were significantly increased by DAPT treatment (Figures 3A and 3B). To confirm the potentiated neurogenesis, qRT-PCR was performed to monitor neural differentiation markers over time. High expression levels of *NGN2*, *TBR2*, *TUBB3*, and *TBR1* were observed in the DAPT-treated samples on NDs 13 and 15. In contrast, the level of *HES1*, a pivotal mediator of the Notch pathway, was decreased during the same period (Figure 3C).

Based on these results, we further attempted to monitor the neural differentiation of live cells after manipulating the function of the HES1 gene. A previous report demonstrated that the loss of Hes1 function in mouse ESCs strongly enhanced neural production (Kobayashi et al., 2009). Here, we infected undifferentiated dual-reporter cells with a lentivirus encoding HES1 short hairpin RNA (shRNA) (Figure S4). After confirming HES1 KD at the early ND stage (Figure 3D), we monitored fluorescent protein expression during neural induction. Both the G^+^ and R^+^ populations appeared earlier after HES1 KD based on an increased fluorescence intensity, indicating that HES1 KD accelerates neural differentiation (Figures 3E and 3F). A reduction in the level of HES1 may preferentially lead to neural lineages, possibly due to the loss of heterogeneity in both the differentiation timing and fate choice (Kobayashi and Kageyama, 2011). These results demonstrate that neural fate conversions induced by drug and gene modulation were validated in real time and that dual-reporter cells can be utilized for screening strategies.

### Tracking cell-migration patterns during neural differentiation

**Figure 4:**
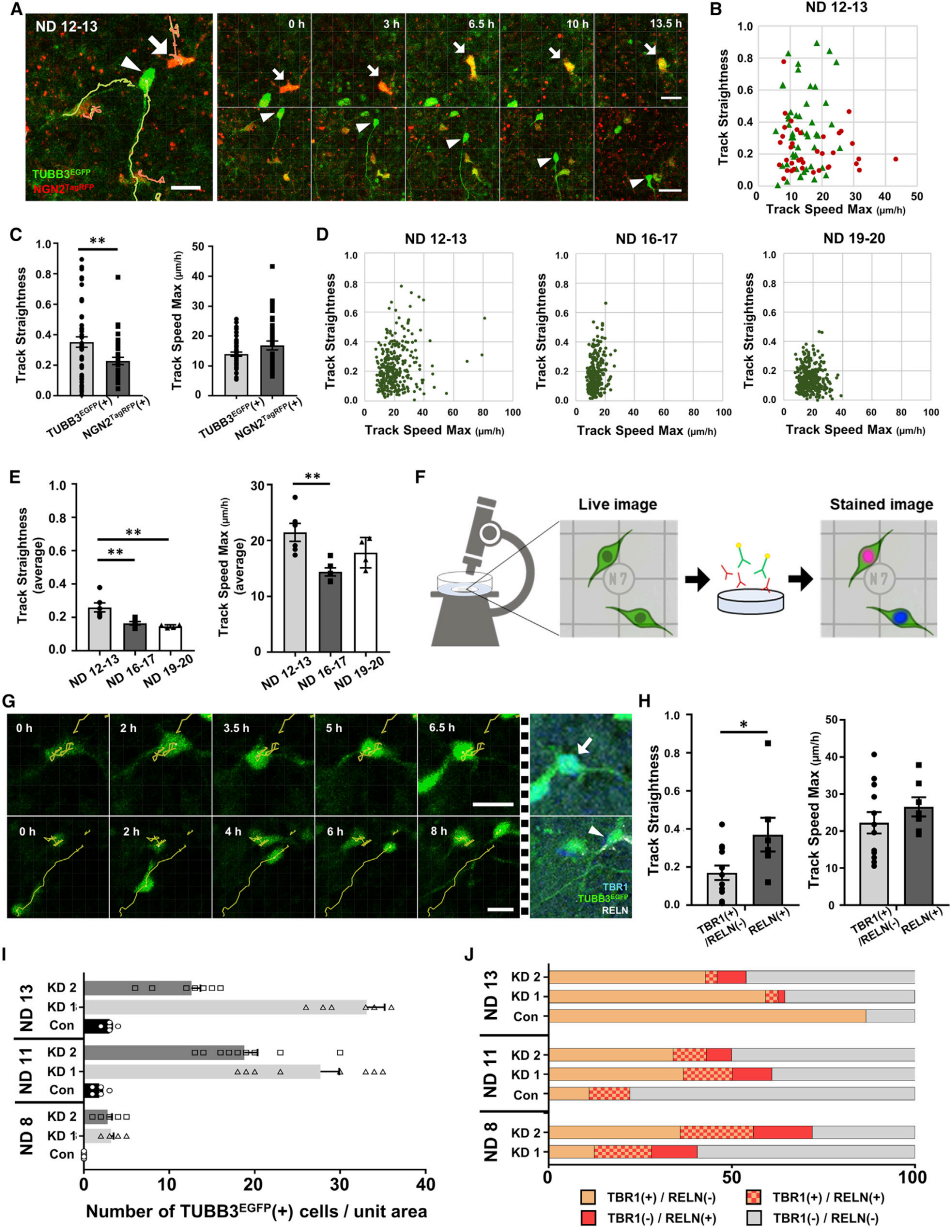
Cell-migration analysis of neural differentiation in correlation with RELN expression (A) Sequential images of cell-fate transition and neural migration (NDs 12–13, T1). Note that R^+^ occasionally converted into G^+^/R^+^ cells during the recording (arrow). G^+^ cells tended to show monodirectional migration (arrowhead). Bar: 20 μm. (B) Quantification of the TS and TSM values for G^+^ (triangle) and R^+^ (circle) cells (NDs 12–13, T1, merge of 3 IEs). (C) Statistic analysis of the data in (B). (D) Differential migration patterns of G^+^ cells from NDs 12–13, 16–17, and 19–20. Approximately 300 tracked cells from 5 IEs at each stage are presented. (E) Statistic analysis of the data in (D). (F) Schematic of subtype specification by immunostaining after live imaging. (G) Sequential tracking images of migrating G^+^ cells that were TBR1^+^/RELN^−^ (arrow) and RELN^+^/TBR1^−^ (arrowhead). The dashed bar indicates the time point of fixation. Bar: 20 μm. (H) Statistical analysis of migration parameters for TBR1^+^/RELN^−^ and RELN^+^ cells (NDs 12–13, T1, 3 IEs). (I) Number of G^+^ cells per unit area on NDs 8, 11, and 13 after HES1 KD (T1, 3 IEs). (J) Ratios of TBR1^+^ and RELN^+^ cells after HES1 KD in (I).

Intensive migration activity is a key feature of neural cells. Although numerous studies on neural migration have been performed using animal models, the migration mechanisms of human neural cells must be elucidated to further understand the expanded cerebral cortex in humans. Here, we characterized the migration pattern of reporter-expressing cells by time-lapse recording followed by quantitative tracking of fluorescent signals. Occasionally, we observed a transition from R^+^ to G^+^ cells at the single-cell level (Figure 4A; Video S2). We compared the characteristics of migration using track straightness (TS) and track speed max (TSM) parameters, defined by the degree of monodirectional migration and the highest speed during the recording period, respectively. We found that G^+^ exhibited a higher TS value than R^+^ cells from ND 12 to 13 (Figures 4B and 4C). This result suggests that the migration mode of human neurons is altered after exit from the neurogenic state, as observed in the embryonic mouse cortex (Tabata and Nakajima, 2003).

Video S2. Time-lapse movie file for Figure 4A (12 min/frame)

To examine the migration ability of neurons depending on the neural induction period, we compared numerous tracking records of G^+^ cells starting on NDs 12, 16, and 19 (Figures 4D and 4E). Importantly, distinct populations had higher TS and TSM values from NDs 12 to 13 than at the later stages. This finding prompted us to further elucidate migration abilities among early-born neurons. During human cortical development, RELN-positive Cajal-Retzius (CR) cells appear as early as 5 gestational weeks (Meyer et al., 2000). The subpopulation of CR cells originating from the pallium is double positive for RELN and TBR1 (Hevner et al., 2003; Meyer and Gonzalez-Gomez, 2018). To investigate the correlation between neural migration pattern and subtype among early-born neurons, we conducted cellular tracking followed by immunostaining with RELN and TBR1 antibodies (Figure 4F). Notably, the migration modes of TBR1 single- and RELN-positive G^+^ cells were compared, and the latter had a higher TS (Figures 4G and 4H), possibly explaining the phenomenon of long-distance migration during cortical formation.

Next, we explored whether the proportion of RELN^+^ cells was altered in ectopic neurons induced by HES1 KD. We found that the earliest G^+^ cells appeared at ND 8 after HES1 KD, while essentially no population appeared in the control group (Figure 4I). Strikingly, RELN^+^ cells accounted for 28% and 36% of HES1 KD1 and KD2 cells, respectively, at ND 8 (Figure 4J). Among them, approximately half of the populations were double positive for TBR1 and RELN, a feature of pallium-derived CR cells. Together, the dual-reporter cells enabled the characterization of the migration mode of early-born neural cells in correlation with their subtypes and revealed the role of HES1 in the efficient production of RELN-positive neurons in the human neural lineage.

CR cell numbers are reported to be increased in the embryonic mouse brain during the early neurogenic stage upon Hes1/3/5 triple knockout (Imayoshi et al., 2008), suggesting the existence of a common mechanism for the production of RELN-positive neurons regulated by the Hes family in the mammalian neural lineage. Clinically, RELN is known to have several neural functions, and its loss may induce brain disorders. Notably, CR cells serve as the main source of Reelin in the developing brain, while Reelin in the adult brain is produced by a subpopulation of GABAergic interneurons, indicating that Reelin may have differential functions in developing and adult brains (Ishii et al., 2016). In the developing brain, Reelin plays important roles in not only the formation of cortical layers, as shown by classical studies on reeler mice, but also neural migration and signaling pathways (Santana and Marzolo, 2017). In addition, the mutation of RELN can cause neuropsychiatric disorders, such as schizophrenia and autism. Here, HES1 KD significantly increased the proportion of RELN-positive neurons. The induction method can be assessed using patient-derived hiPSCs to characterize RELN dysfunction both *in vitro* and *in vivo* after transplantation in model animals.

## Experimental procedures

### hiPSC lines

Use of human-derived material in the research is approved by the Research Ethics Committee of KBRI (KBRI-201603-BR-001-01). The hiPSC line used in this study (RPChiPS771, ReproCELL, Yokohama, Japan) was characterized in a previous publication (Iwashita et al., 2019). Briefly, hiPSCs were obtained by the self-replicative RNA reprogramming method. Pluripotency toward three germ layers and higher efficiency toward neural lineage compared with other established hiPSC lines (1231A3, 1383D2, and 1383D6, CiRA, Kyoto University) were confirmed by qRT-PCR.

### Statistical analysis

Prism (GraphPad Software) was used for statistical analyses. Differences between two groups were analyzed by the two-tailed Student’s t test, and differences between more than two groups were analyzed by ANOVA followed by Tukey’s test. Differences were considered significant for p values <0.05 (^∗^p < 0.05, ^∗∗^p < 0.01, and ^∗∗∗^p < 0.001). All error bars represent the SEM. For qRT-PCR analysis, each point indicates the averaged value of triplicated reactions.

### Other materials and methods

All animal protocols were approved by the KBRI institutional animal care and use committee (IACUC-20-00044). Other materials and methods are described in the supplemental experimental procedures.

## Author contributions

Study design and conceptualization, G.P. and Y.K.; manuscript preparation, G.P. and Y.K.; experiments, G.P. for all, M.S. for imaging of cell divisions, and W.L. for transplantation; resources, A.H. and T.K.; project administration, Y.K.

## Supplemental information

Document S1. Figures S1–S4, Table S1, and Supplemental experimental procedures

Document S2. Article plus supplemental information
