# Supplementary material for: Direct visualization of the transition status during neural differentiation by dual-fluorescent reporter human pluripotent stem cells
Source: Stem Cell Reports. 2022 Aug 4;17(9):1903–13. doi: 10.1016/j.stemcr.2022.07.001 (PMC9481873; doi:10.1016/j.stemcr.2022.07.001)
Supplement: Document S1. Figures S1–S4, Table S1, and Supplemental experimental procedures [file mmc1.pdf]

Stem Cell Reports, Volume *17*

Supplemental Information

Direct visualization of the transition status during neural differentiation by dual-fluorescent reporter human pluripotent stem cells

Gwanghyun Park, Minkyung Shin, Wonyoung Lee, Akitsu Hotta, Taeko Kobayashi, and Yoichi Kosodo

Supplemental information

Supplemental Figures

A

RGEN target for TUBB3	Chromosome	Position	Direction	GC contents (%)	Mismatches			Target name
					0	1	2	
CGAGATGTACGAAGACGACGAGG	Chr16	89935752	+	55.0	1	0	0	G2
GTACATCTCGCCCTCTTCCTCGG	Chr16	89935739	-	55.0	1	0	0	G3
GATGTACGAAGACGACGAGGAGG	Chr16	89935523	-	65.0	1	0	1	G4
**RGEN target for NGN2**	**Chromosome**	**Position**	**Direction**	**GC Contents (%)**	**Mismatches**			**Target name**
					**0**	**1**	**2**	
CCGGGTCAGACATGGACTATTGG	Chr4	112514722	-	55.0	1	0	0	T1
CACTTTATCGCCCGCCAGCCCGG	Chr4	112514745	-	65.0	1	0	0	T2
TAGATACAATCCCTGGCTATGGG	Chr4	112514657	+	40.0	1	0	0	T3
TGGGGAGGTGAGGTGCATAGCGG	Chr4	112514676	+	60.0	1	0	0	T4
GCAGCTCTAGATACAATCCCTGG	Chr4	112514650	-	50.0	1	0	2	T5

B

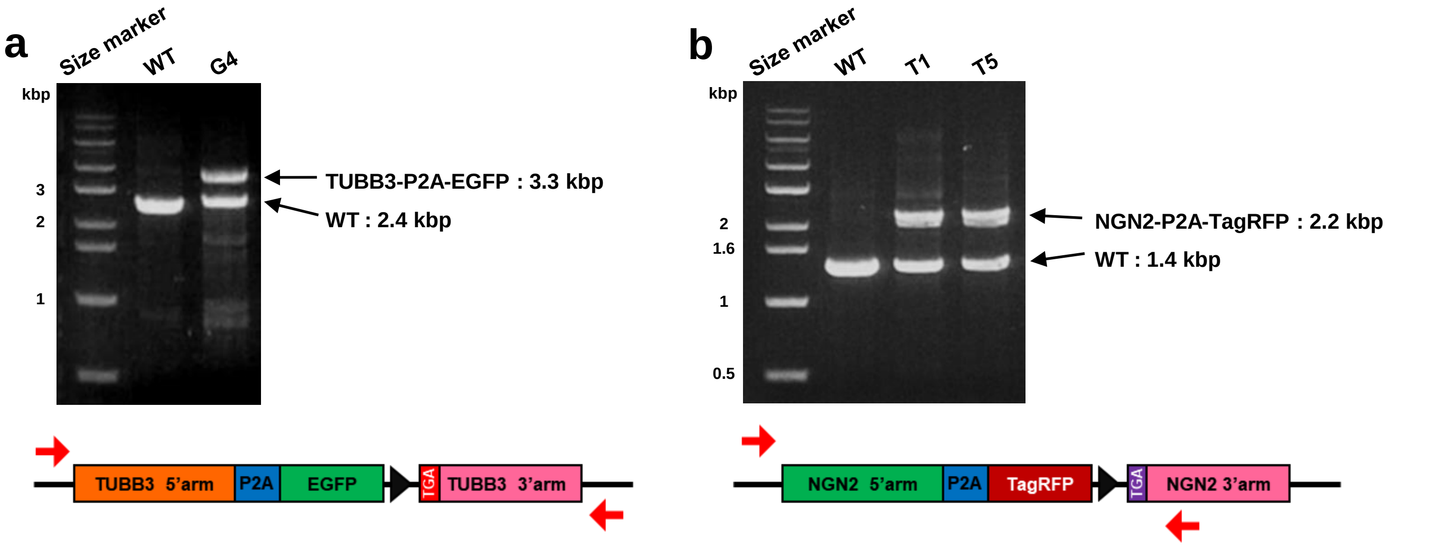

C

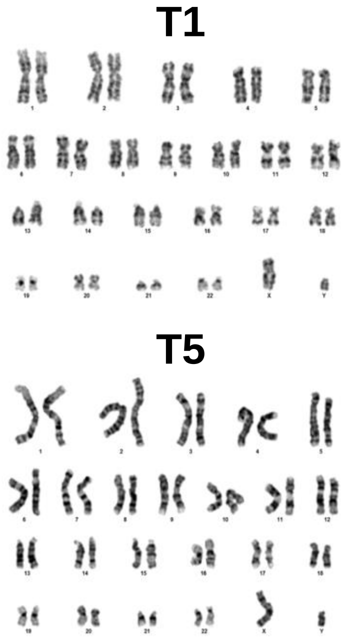

D

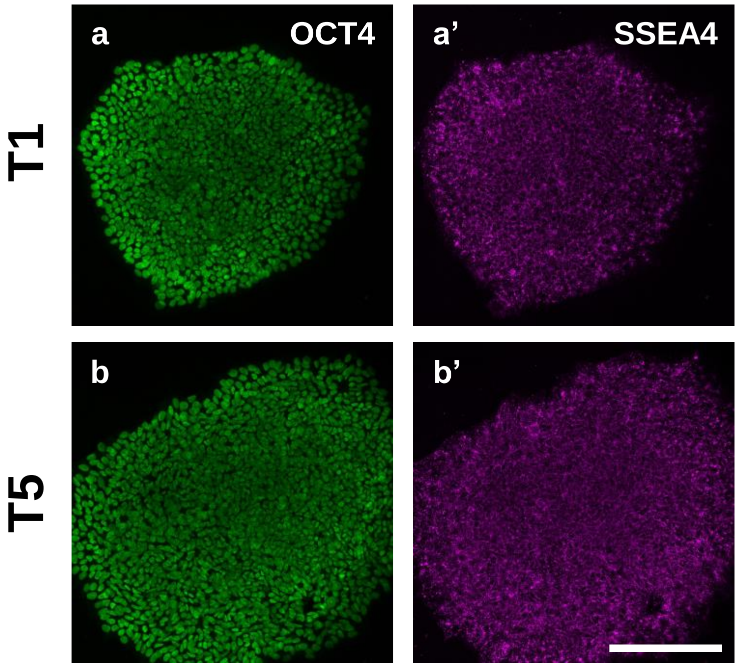

Supplemental Figure 1. Construction of TUBB3^{EGFP} and NGN2^{TagRFP} dual-reporter hiPSCs, Related to Figure 1A

A. Sequences of the sgRNAs used to construct TUBB3^{EGFP} and NGN2^{TagRFP} .

B. Confirmation of heterozygous KI by PCR. The arrows indicate the positions of the primers (a, TUBB3-CRISPR-confirm-F1 and TUBB3-CRISPR-confirm-R2; b, NGN2-genome-F1 and NGN2-CRISPR-confirm-R3, each primer sequence is indicated in Table S1).

C. Karyotype analysis of dual-reporter hiPSCs (T1 and T5)

D. Immunostaining images obtained using antibodies for OCT4 (green) for T1 (a) and T5 (b) and SSEA4 (magenta) for T1 (a') and T5 (b') fixed at the pluripotent stage. Bar = 200 μm.

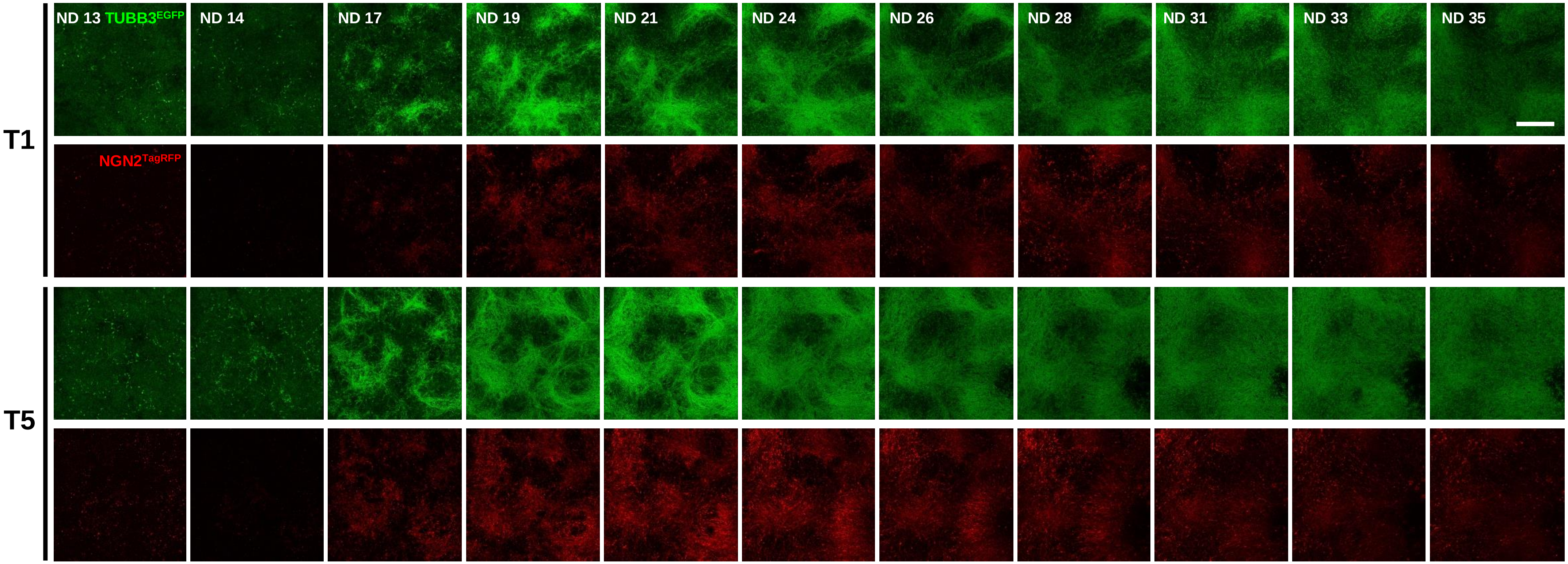

Supplemental Figure 2. Sequential images of $TUBB3^{EGFP}$ and $NGN2^{TagRFP}$ expression in dual-reporter cells, Related to Figure 1C
Sequential images of $TUBB3^{EGFP}$ and $NGN2^{TagRFP}$ expression in dual-reporter cells (T1 and T5) during neural differentiation (from ND 13 to 35) at the same position on the grid culture dish. Bar = 300 µm.

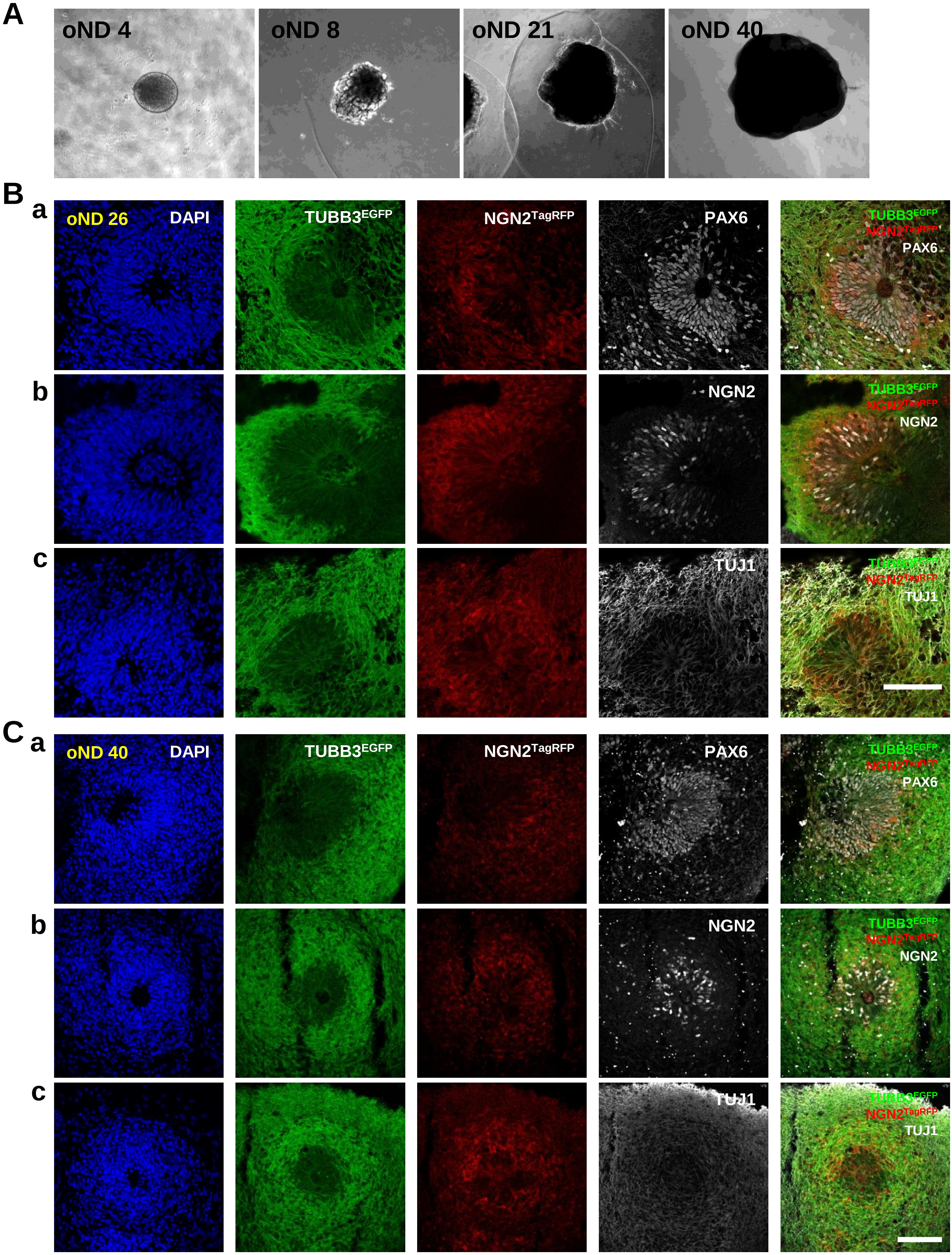

Supplemental Figure 3. Cerebral organoids generated from dual-reporter hiPSCs, Related to Figure 2A

A. Complete views of cerebral organoids on oND 4, 8, 21, and 40.

B. Immunostaining images of organoids obtained using antibodies against PAX6 (a), NGN2 (b), and TUJ1 (c) on oND 26.

C. Immunostaining images of organoids obtained using antibodies against PAX6 (a), NGN2 (b), and TUJ1 (c) on oND 40.

Bars = 100 µm.

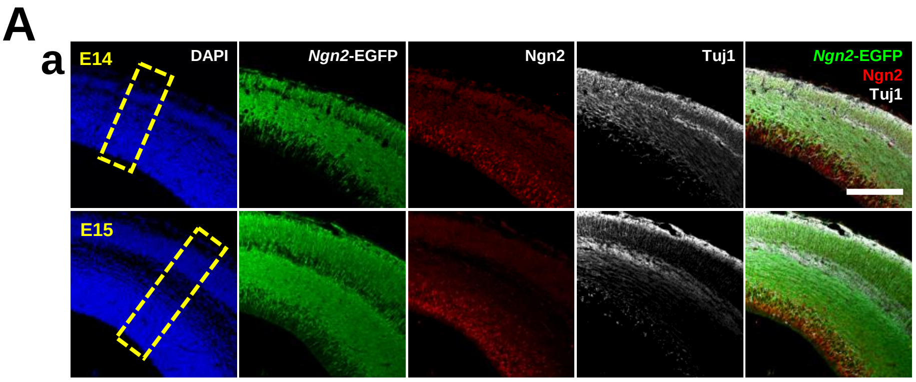

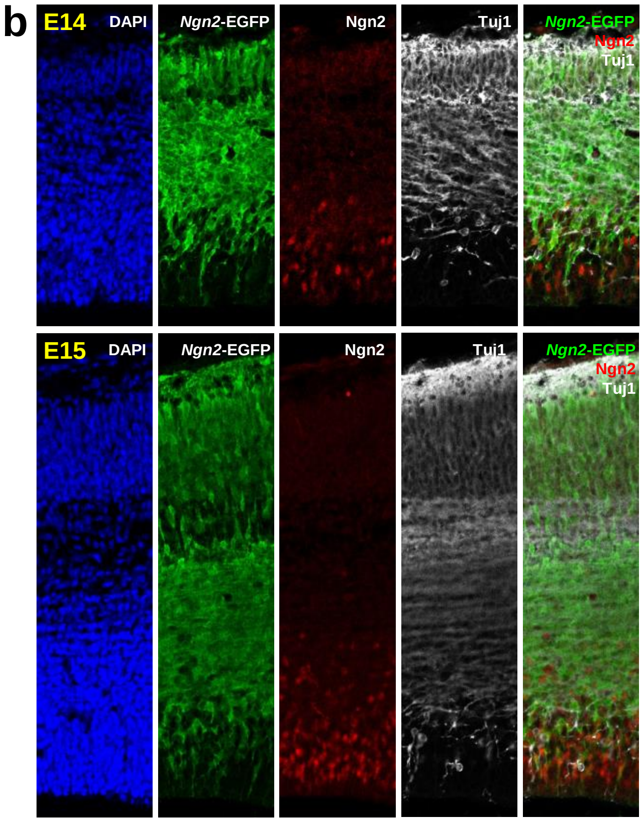

B

ATCACACAGGATCCGGAGCTGGTGCTGATAACAGCGGAATCCCCGTCTACCTCTCTCCTTGGTCCTGGAACAGCGCTACTGATCACCAAGTAG
CCACAAAATATAATAAACCCTCAGCACTTGCTCAGTAGTTTTGTGAAAGTCTCAAGTAAAAGAGACACAAACAAAAAATTCTTTTTCGTGAAGAAC
TCCAAAAATAAAATTCTCTAGAGATAAAAAAAAAAAAAAAAGGAAAATGCCAGCTGATATAATGGAGAAAAATTCCTCGTCCCCGGTGGCTGCTA
CCCCAGCCAGTGTCAACACGACACCGGATAAACCAAAGACAGCATCTGAGCACAGAAAGTCATCAAAGCCTATTATGGAGAAAAGACGAAGAGC
sh4
AAGAATAAATGAAAGTCTGAGCCAGCTGAAAACACTGATTTTGGATGCTCTGAAGAAAGATAGCTCGCGGCATTCCAAGCTGGAGAAGGCGGAC
ATTCTGGAAATGACAGTGAAGCACCTCCGGAACCTGCAGCGGGCGCAGATGACGGCTGCGCTGAGCACAGACCCAAGTGTGCTGGGGAAGTA
sh5
CCGAGCCGGCTTCAGCGAGTGCATGAACGAGGTGACCCGCTTCCTGTCCACGTGCGAGGGCGTTAATACCGAGGTGCGCACTCGGCTGCTCG
GCCACCTGGCCAACTGCATGACCCAGATCAATGCCATGACCTACCCCGGGCAGCCGCACCCCGCCTTGCAGGCGCCGCCACCGCCCCCACCG
GGACCCGGCGGCCCCCAGCACGCGCCGTTCGCGCCGCCGCCGCCACTCGTGCCCATCCCCGGGGGCGCGGCGCCCCCTCCCGGCGGCGC
CCCCTGCAAGCTGGGCAGCCAGGCTGGAGAGGCGGCTAAGGTGTTTGGAGGCTTCCAGGTGGTACCGGCTCCCGATGGCCAGTTTGCTTTCC
TCATTCCCAACGGGGCCTTCGCGCACAGCGGCCCTGTCATCCCCGTCTACACCAGCAACAGCGGCACCTCCGTGGGCCCCAACGCAGTGTCA
sh8
CCTTCCAGCGGCCCCTCGCTTACGGCGGACTCCATGTGGAGGCCGTGGCGGAACTGAGGGGGCTCAGGCCACCCCTCCTCCTAAACTCCCCA
ACCCACCTCTCTTCCCTCCGGACTCTAAACAGGAACTTGAATACTGGGAGAGAAGAGGACTTTTTTGATTAAG TGGTTACTTTGTGTTTTTTTAAT
sh9 sh10 sh6
TTCTAAGAAGTTACTTTTTGTAGAGAGAGCTGTATTAAGTGACTGACCATGCACTATATTTGTATATATTTTATATGTTCATATTGGATTGCGCCTTT
sh7 sh1 sh2 sh3
GTATTATAAAAGCTCAGATGACATTTCGTTTTTTACACGAGATTTCTTTTTTATGTGATGCCAAAGATGTTTGAAAATGCTCTTAAAATATCTTCCTT
TGGGGAAGTTTATTTGAGAAAATATAATAAAAGAAAAAAGTAAAGGCAAAAAAAAAAAAAAAAA

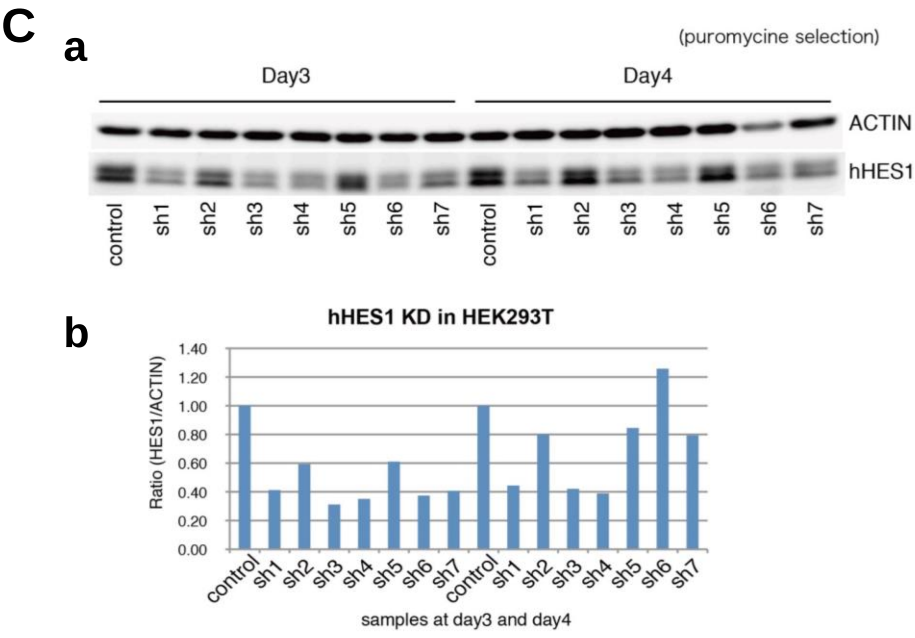

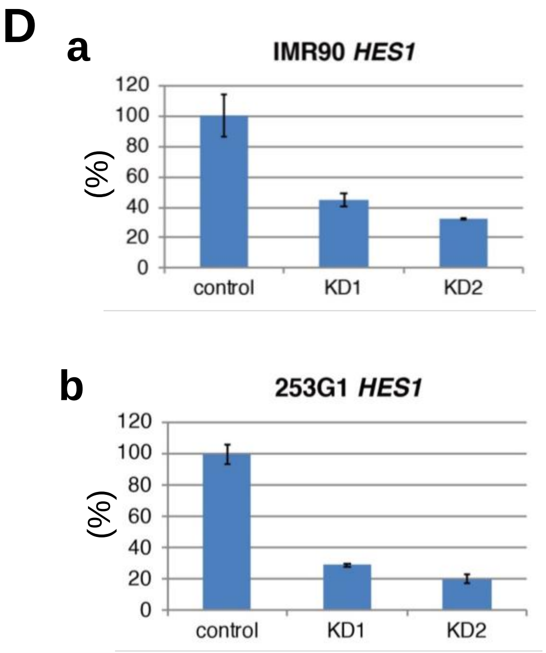

Supplemental Figure 4. Expression of *Ngn2*-EGFP in the developing mouse brain, Related to Figure 2A and confirmation of HES1 KD in HEK293T cells and other hiPSC lines, Related to Figure 3D

A. Immunostaining images of embryonic brain sections of *Ngn2*-EGFP mice obtained using antibodies against Ngn2 (red) and Tuj1 (white) at E14.5 and E15.5. Bar = 200 µm.

B. Candidate shRNA sequences for HES1 KD. Note that sh3 = KD1 and sh4 = KD2.

C. Western blotting to quantify the endogenous HES1 expression in HEK293T cells at 3 and 4 days after the transfection of KD constructs.

D. qRT–PCR analyses of HES1 in IMR90 (a) and 253G1 (b) hiPSC lines after the transfection of two different shRNA constructs (KD1 and KD2) at the pluripotent stage. The expression level was normalized to that in the control.

Supplemental Movie Legends

Movie S1
Time-lapse movie file for Figure 1D (12 min/frame)

Movie S2
Time-lapse movie file for Figure 4A (12 min/frame)

Table S1. Primer and antibody list

Primers for qRT-PCR		
Gene	Forward primer	Reverse primer
GAPDH	CGCTCTCTGCTCCTCCTGTT	CCATGGTGTCTGAGCGATGT
HES1	CGGACATTCTGGAAATGACA	TACTTCCCCAGCACACTTGG
NGN2	ACCATCTCACTTCACAGGGCAG	TCAAGAAGCGCCCCCAAAAC
TBR2	CACCGCCACCAAACTGAGAT	CGAACACATTGTAGTGGGCAG
TBR1	ATGGGCAGATGGTGGTTTTA	GACGGCGATGAACTGAGTCT
TUBB3	CCTGGAACCCGGAACCAT	AGGCCTGAAGAGATGTCCAAAG
Albumin	TTTGCAGATGTCAGTGAAAGAGA	TGGGGAGGCTATAGAAAATAAGG
WPRE	GTCCTTTCCATGGCTGCTC	CCGAAGGGACGTAGCAGA

Primers for knock-in generation and confirmation		
Primer	Sequence	Experiment
TUBB3-5'-fwd-1	AAAGCTGGGTCTAGAGTCAGAGTGGGGCCGGCA	TUBB3-EGFP knock-in donor DNA cloning
G4-rev-1	AGTAGCTCCGCTTCCCTTGGGGCCCTGGGCCTCCGACTCTTC	
2A-EGFP-fwd-1	GCCCAGGGCCCCAAGGGAAGCGGAGCTACTAACT	
EGFP-rev-1	TTTAAACGCGGCCGCTTACTTGTACAGCTCGTC	
TUBB3-3'-fwd-1	CGAAGTTATGAATTCTGAAGCTGCTCGCAGCTGGA	
TUBB3-3'-rev-1	ACCAATTCAGTCGACCGGCCTCTCTGGGCTCCA	
sgRNA-G4-fwd	GAGACCACTTGGATCCGATGTACGAAGACGACGAGGGTTTTAGAGCTAGAAATAGCA	TUBB3-EGFP knock-in guide RNA cloning
sgRNA-Universal-rev	GCCCGGGTTTGAATTCAAAAAAAGCACCGACTCGGTGCCACTTTTTCAAGTTGATAACGGACTAGCCTTATTTTAACTTGCTATTTCTAGCTC	
TUBB3-CRISPR-confirm-F1	GTCTGGACTGCAGAGTCCCTGG	TUBB3-EGFP knock-in genotyping and sequencing
EGFP-reverse-R1	GACACGCTGAACTTGTGGCCG	
Puro-confirm-F2	GCAACCTCCCCTTCTACGAG	
TUBB3-CRISPR-confirm-R2	GCAGAAGGATCCAGCTAACACGCTG	
TUBB3-CRISPR- confirm-F3	TTGCGTGTGGCAGTTTTAAG	
TUBB3-CRISPR- confirm-R3	AAGGACATCTCTGGCACCAA	
NGN2-5'arm-F-1	AAAGCTGGGTCTAGAGATGTTCGTCAAATCCGAGA	NGN2-TagRFP knock-in donor DNA cloning
NGN2-5'arm-R	AGTAGCTCCGCTTCCGATACAATCCCTGGCTATGGGGAGGTGAGGTGCATAGCGGTGCTTGTCGGGAGGTGGGGGCTGCCAATAGTCCATGTCCGATCCCGCCGGGCTAGCGGGTGA TAAGGTGCA	
2A-RFP-F	GCCAGGGATTGTATCGGAAGCGGAGCTACTAACTTCAGCCTGCTGAAGCAGGCTGGAGACGTGGAGGAGAACCCTGGACCTATGGTGTCTAAGGGCGAAG	
2A-RFP-R-1	TTTAAACGCGGCCGCTCAATTAAGTTTGTGCCCCA	
NGN2 3'arm-F	CGAAGTTATGAATTCTAGAGCTGCCATTTCTGCTA	
NGN2 3'arm-R	CAGTCGACTGGATCCTGCTTAGGTTTTCAGACTGT	
sgRNA-Target1-fwd	GAGACCACTTGGATCCCCGGGTCAGACATGGACTATGTTTTAGAGCTAGAAATAGCA	NGN2-TagRFP knock-in guide RNA cloning
sgRNA-Target5-fwd	GAGACCACTTGGATCCGCAGCTCTAGATACAATCCCGTTTTAGAGCTAGAAATAGCA	
NGN2-CRISPR-confirm-fwd-1	GCAGCTGGATGGCAAATAAT	NGN2-TagRFP knock-in genotyping and sequencing
NGN2-CRISPR-confirm-rev-1	TCAGCTCTTCGCCCTTAGAC	
NGN2-CRISPR-confirm-fwd-2	GCAACCTCCCCTTCTACGAG	
NGN2-CRISPR-confirm-rev-2	CTGATAGTGACCTGTTCGTTGC	
NGN2-CRISPR-confirm-F3	AATTCCACCTCCCCCTACAG	
NGN2-CRISPR-confirm-R3	GCCCGTCTGAATGAAGGATA	
hEF1a-F1	GACGGGGGTAGTCTCAAGC	
hEF1a-R1	CTTTCCGCTCACGCAACT	
NGN2-genome-F1	AGCCACCGCTTCCAACTAC	

Antibodies							
Antibody	Company	Catalog number	Dilution	Antibody	Company	Catalog number	Dilution
GFP	Aves	GFP-1010	1:1000	BrdU	Abcam	ab6326 (BU1/75)	1:1000
RFP	Evrogen	AB233	1:1000	anti-chicken Alexa488	Jackson Immuno Research	703-545-155	1:1000
TUJ1	Biolegend	MMS-435P	1:1000	anti-rabbit Alexa555	Invitrogen	A31572	1:1000
NGN2	CST	13144	1:200	anti-mouse Alexa647	Invitrogen	A31571	1:1000
KI67	BD	556003	1:1000	anti-rabbit Alexa647	Invitrogen	A31573	1:1000
PAX6	MBL	PD022	1:1000	anti-rabbit Alexa647	Invitrogen	A21245	1:1000
TBR1	Abcam	ab31490	1:1000	anti-rat Alexa647	Invitrogen	A21247	1:1000
CTIP2	Abcam	ab18465	1:1000	anti-rabbit Alexa405	Invitrogen	A48258	1:1000
SATB2	Abcam	ab51502	1:1000	anti-goat Alexa647	Invitrogen	A21447	1:1000
RELN	R&D	AF3820	1:100				

Supplemental experimental procedures

Animals

Pregnant ICR mice were purchased from Core Tech, South Korea. *Neurog2*-EGFP transgenic mice were obtained from the GENSAT project (#000266-UNC) and bred at KBRI by crossing with C57BL/6 mice. The day of birth was defined as postnatal day 0 (P0).

Neural differentiation

The expansion and neural differentiation of hiPSCs were performed using a previously described method (Espuny-Camacho et al., 2013) with some modifications. HiPSCs were maintained in StemFit Basic 04 medium (Ajinomoto, AJ200B, Japan) in a multiwell dish (Corning, USA) coated with Vitronectin XF (Stemcell Technologies, 07180, Canada). For neural differentiation, cells were plated on Vitronectin XF-coated dishes at a defined density (6.0 x 10^4 cells/well in a 6-well plate) in StemFit Basic 04 medium containing Y-27632 (ROCK inhibitor; 10 μM) (Wako, 036-24023, Japan) two days prior to neural differentiation. On the day of differentiation initiation (defined as "ND 0"), the medium was replaced with neural differentiation medium supplemented with N2 (R&D Systems, AR009, USA), B27 (Gibco, 17054-044, USA) and Noggin (for ND 0-16, 100 ng/ml, R&D Systems, 6057-NG-100, USA) and changed every 2 days. On ND 4 or 5, cells were replated using TrypLE Select CTS (Gibco, A12859-01, USA) diluted in 0.5 mM EDTA (Invitrogen, 15575-038, USA) at a density of 2.0 x 10^5 cells/well in a 6-well plate. The medium was changed every 2 days and included Y-27632 for 2 days after replate and noggin until ND 16. In the DAPT treatment assay, DAPT was added to the medium beginning on ND 11 at a concentration of 5 μM. To evaluate gene expression in hiPSCs, qRT–PCR was performed as described previously (Iwashita et al., 2019) using specific primer sets (Table S1).

Design and cloning of the KI constructs

CRISPR RGEN Tools (Bae et al., 2014) was used to find candidates for single-guide RNA (sgRNA) target sites near the stop codon of the NGN2 or TUBB3 coding sequence and to evaluate off-target scores. Three and five target sites were selected for the $TUBB3^{EGFP}$ and $NGN2^{TagRFP}$ KI constructs, respectively (Figure 1A and S1). To clone a DNA-coding sgRNA sequence into an expression vector, a pair of oligonucleotides (a forward primer containing the sgRNA target sequence and a reverse primer containing an sgRNA scaffold sgRNA) was designed according to a previously described strategy (Li et al., 2016). After PCR amplification using the primer set, the PCR fragment was cloned into pHL-H1-ccdB-mEF1α-RiH (Addgene ID: 60601). To construct donor DNA, P2A-TagRFP, P2A-EGFP, 5' and 3' homology arms of NGN2 and TUBB3 genes were amplified by PCR using genomic DNA extracted from wild type hiPSCs, then ligated by In-Fusion system (Takara, 639648 Japan) into pENTR-DMD-Donor vector (Addgene ID:60605), which has floxed EF1a-Puro cassette. P2A-TagRFP and P2A-EGFP cassettes were inserted just before the stop codon of the coding sequence of NGN2 or TUBB3 genes. The primers and vectors used in this study are listed in Table S1.

Electroporation

To establish dual-reporter cell lines, DNA constructs were sequentially electroporated (EP) into hiPSCs (Figure 1A). The first round of EP was carried out to establish $TUBB3^{EGFP}$ KI cells. A Cas9 expression plasmid (Addgene ID: 60599) (Li et al., 2016), an sgRNA expression and donor plasmids were cotransfected into 1.0 x 10^6 hiPSCs using a NEPA21 electroporator (Nepagene, Japan) under previously described conditions (voltage of 125 V and electroporation pulse width of 5 ms (Oceguera-Yanez et al., 2016)). Immediately after EP, the cells were transferred into medium containing Y-27632 to prevent apoptosis. To select cells carrying the donor DNA sequence, puromycin (Sigma, P9620, USA, 250 ng/ml in medium) was administered 2 to 5 days after EP. After the passaging step, low-cell density culture was performed (300 cells in a well in a 6-well dish) until small colonies were formed, and single colonies were then selected and transferred to the wells of 24-well dishes. After expansion, KI candidate clones were selected by genotyping (Figure S1) using the primer sets (Table S1). Subsequently, the second round of EP was performed with CAG-Cre and CAG-RFP (Shitamukai et al., 2011) to remove the floxed-

puromycin cassette by Cre recombination. Low-cell density culture followed by genotyping by the primer sets (Table S1) allowed the selection of RFP-positive clones excised the puromycin cassette by the Cre plasmid function. After the expansion step, the third round of EP was performed with the sgRNA and the donor DNA sequence to generate NGN2^{TagRFP} KI cells using the method described for the first EP. Two independent clones (T1 and T5) were selected after the neural induction of 4 clones each for T1 and T5, followed by the fourth round of EP to remove the puromycin cassette by the Cre plasmid. All clones were heterozygous KI cells (Figure S1), and successful genome editing was confirmed by sequencing the corresponding genomic loci using specific primer sets (Table S1). Confirmation of karyotypes (Figure S1) was conducted by Samkwang Medical Laboratory (Republic of Korea).

Cerebral organoid generation

Cerebral organoids were generated using a STEMdiff Cerebral Organoid Kit (Stemcell Technology Cat# 08570, Canada) according to the manufacturer's instructions. Briefly, hiPSCs maintained in StemFit Basic 04 were detached by TrypLE Select CTS diluted in 0.5 mM EDTA and transferred to an ultralow attachment 96-well plate (Corning, 7007, USA) at a density of 9.0 x 10^3/well in embryoid body (EB) formation medium containing Y-27632 (10 μM). For mixed organoid generation, 8.1 x 10^3 original hiPSCs and 9.0 x 10^2 TUBB3^{EGFP}/NGN2^{TagRFP} reporter hiPSCs were mixed and transferred into each well. The medium was changed every 2 days without adding Y-27632. To induce cerebral organoid formation, 1 or 2 EBs were moved to a 24-well ultralow attachment plate (Corning, 3473, USA) containing induction medium. After 7 days, organoids were-embedded in Matrigel (Corning, 354230, USA) and cultured with Expansion medium. After 10 days, the organoids were matured on an orbital shaker inside a CO_2 incubator and cultured for up to 40 days. BrdU (Sigma, B9285, USA, 1 μM in medium) was added on oND 26 cells for 4 h before fixation. For double labeling, EdU (Invitrogen, C10340, USA, 4 μM) was added to the cells for 4 h on oND 26, and the cells were cultured after washing with medium. After 2 weeks, BrdU was added on oND 40 for 4 h prior to fixation.

Transplantation

Transplantation of hiPSC-derived neural cells to the developing mouse brain was performed using the highly efficient transplantation method published previously (Nagashima et al., 2014) with slight modifications. Briefly, neural differentiation from dual-reporter hiPSCs was conducted using the 2D culture method. On ND 20 and 27, cells were detached from the culture dish using Accutase (Gibco, A1110501, USA) and used to make a 50 μl mixed solution containing (1.0x10^5 cells/μl) donor cells, 20 mM ethylene glycol tetraacetic acid (EGTA), 10 μM Y-27632, and 0.01% Fastgreen. One to two microliters of the mixed solution was injected into the lateral ventricles of E14.5 ICR mice *in utero*, and neonatal brains were then dissected and fixed with 4% paraformaldehyde (PFA) at P0. To observe the donor cells in the host brain tissue, 100-μm vibratome sections were prepared and subjected to immunostaining analysis using antibodies against GFP (Aves, GFP-1010, USA) and RFP (Evrogen, AB233, Russia) to enhance the signals of TUBB3^{EGFP} and NGN2^{TagRFP}, respectively. Before observing the stained vibratome sections, CUBIC treatment was performed for tissue clearing. CUBIC was prepared according to a previous study (Susaki et al., 2014), and stained tissue sections were immersed in 100 μl for 5-10 min with gentle shaking. Fluorescence 3D images were acquired by confocal microscopy (Andor Technology, Dragonfly 502w, UK) using 488 and 561 nm lasers. Z-stack sections were acquired at 0.5 μm intervals between each optical section using a 40x objective lens.

KD of HES1 function

To make the DNA construct, the following oligos containing the target sequence for human HES1 were inserted into the BbsI site of the psiRNA-h7SKneo plasmid (InvivoGen, USA): ACCTC-GGGTTGTTACtTAAGTCCTgGA-TCAAGAG-TCTAGGACTTAGGTAACATCC-TT (control scramble), ACCTC-GAtCATGCgCTgTATTTGTAT-TCAAGAG-ATACAAATATAGTGCATGGTC-TT (HES1-sh3 for KD1), and ACCTC-GCgTCTGgGCgCAGAAAGTCA-TCAAGAG-TGACTTTCTGTGCTCAGATGC-TT (HES1-sh4 for KD2). The ACCTC on the 5' side and the TT on the 3' side were added to generate the restriction enzyme site, and the TCAAGAG sequence at the center was added to generate the loop for the short hairpin. To prevent mutation during replication, 3 point mutations (indicated by lowercase letters) were inserted. The sequence

containing both the h7SK promoter and inserts (h7SK-shRNA) was then transferred to the CSII vector containing PGK-neo-pA. All h7SK-shRNAs were inserted into PGK-neo-pA in CSII at the 3' side. To produce lentivirus for HES1 KD, HEK293T cells were plated in DMEM/F12 supplemented with 10% FBS and 1% penicillin/streptomycin (3.0 x 10^6 cells on a 10 cm dish) 24 h before transfection. HEK293T cells were cotransfected with a CSII vector containing the KD constructs (8.4 μg), psPAX2 (6.2 μg) and pMD2. G (2.6 μg) using PEI MAX (Polysciences, 24765, USA) for lentivirus packaging. Media containing forskolin (Wako, 067-02191, Japan, 10 μM) were changed at 8 h after transfection. Media containing lentivirus were collected at 54 h after transfection, passed through a 0.45 μm filter and concentrated by centrifugation. For titration, 5.0 x 10^4 HEK293T cells were infected with serial dilutions of lentivirus in a well of a 12-well plate. Genomic DNA was extracted 4 days after infection, and the titer was measured by qRT–PCR (Barczak et al., 2015). $TUBB3^{EGFP}/NGN2^{TagRFP}$ reporter hiPSCs were infected with lentivirus at an MOI of 1 after dissociation and selected with 250 μg/ml G418 (Roche, 4727878001, Switzerland) for 24 h after 4-6 days.

Immunostaining
Cells cultured on cover glass were fixed with 4% PFA for 10 min. Fixed samples were permeabilized with 0.5 x Triton X-100 in PBS for 10 min and then incubated in blocking solution (2% BSA in PBS) for 1 h at room temperature. Subsequently, samples were incubated with primary antibodies diluted in the blocking solution for 2 h at room temperature or overnight at 4°C. After rinsing with PBS, fluorochrome-conjugated secondary antibodies and DAPI diluted in PBS were applied for 1 h at room temperature. Samples were mounted with a drop of mounting medium (Invitrogen, P36961, USA) and observed by confocal microscopy (Leica, SP8, Germany). Organoids were fixed with 4% PFA overnight at 4°C and then with 20% sucrose solution overnight at 4°C. Organoids were initially embedded in gelatin/sucrose solution (Lancaster and Knoblich, 2014) and then re-embedded in OCT compound (SAKURA, 4583, Japan) to make a frozen block. The tissue blocks were sectioned at a thickness of 20 μm for immunostaining. Information regarding the antibodies used in this study is provided in Table S1.

Live cell imaging
To record the transient expression of fluorescent reporters during neural differentiation for several weeks, snapshot imaging of EGFP and TagRFP was performed on cells cultured on a glass bottom dish with grid lines (IWAKI, 3922-035, Japan). Using the grid line as a landmark, it was possible to find cells in the same position under the bright field of the confocal microscope on consecutive days. For live imaging of cell division, a glass bottom dish without grid lines (Nunc, 150682, USA) was used. To maintain the cell survival environment during microscope imaging, the incubation chamber (LCI, Stage-top Incubator System TC, Korea) was set at 5% CO_2 and 37°C. Cells were maintained in a conventional CO_2 incubator when not being imaged. To track the cell division and migration of cells expressing fluorescent reporters, time-lapse imaging was performed with a confocal microscope (Leica, SP8, Germany) for 12-15 h at 10-15 min intervals in the incubation chamber.

Image analysis
The fluorescence intensities of EGFP and TagRFP were quantified using ImageJ software (https://imagej.nih.gov/ij/download.html). The background was removed by the subtract background tool, and the rolling ball radius parameter was set to at least the size of the cell that was not part of the background. The threshold was adjusted to distinguish the components of interest and the background. The intensity was measured automatically based on the threshold images. Sholl analysis of differentiating cells was performed using the Simple Neurite Tracer (SNT) function in ImageJ as previously described (Binley et al., 2014). Sholl analysis was performed using the tracing images, and concentric rings spaced 3.5 μm apart centered on the soma were applied.

Cell migration analysis
Time-lapse recordings of cell migration during neural differentiation were analyzed using Imaris software (Oxford Instruments, x64 9.0.2, UK). Cells were marked as spots using the Spot tool, and the migration of each cell was tracked over time automatically. For the filter type, “quality for

classify spots” was selected, and the lower threshold parameter was set to 8.00. The autoregressive motion algorithm was selected using 20 μm for the MaxDistance and 3 μm for the MaxGapSize settings. Statistical values of track straightness (TS) and track speed max (TSM) were utilized to characterize the cellular migration pattern.

Cell division analysis
Fluorescent signal intensity of time-lapse recorded images including cell division were analyzed using ImageJ software. G^+/R^- neurons identified in the analyzed frame were considered mature neurons. The fluorescent signal was divided by the area calculated by outlining cell bodies of mature neurons or dividing cells. The EGFP signal of G^+/R^- neurons was set as the normalization standard (100%) to calculate relative intensity for dividing cells.

Supplemental Information References

Bae, S., Park, J., and Kim, J.S. (2014). Cas-OFFinder: a fast and versatile algorithm that searches for potential off-target sites of Cas9 RNA-guided endonucleases. Bioinformatics *30*, 1473-1475.

Barczak, W., Suchorska, W., Rubis, B., and Kulcenty, K. (2015). Universal real-time PCR-based assay for lentiviral titration. Mol Biotechnol *57*, 195-200.

Binley, K.E., Ng, W.S., Tribble, J.R., Song, B., and Morgan, J.E. (2014). Sholl analysis: a quantitative comparison of semi-automated methods. J Neurosci Methods *225*, 65-70.

Espuny-Camacho, I., Michelsen, K.A., Gall, D., Linaro, D., Hasche, A., Bonnefont, J., Bali, C., Orduz, D., Bilheu, A., Herpoel, A.*, et al.* (2013). Pyramidal neurons derived from human pluripotent stem cells integrate efficiently into mouse brain circuits in vivo. Neuron *77*, 440-456.

Iwashita, M., Ohta, H., Fujisawa, T., Cho, M., Ikeya, M., Kidoaki, S., and Kosodo, Y. (2019). Brain-stiffness-mimicking tilapia collagen gel promotes the induction of dorsal cortical neurons from human pluripotent stem cells. Sci Rep *9*, 3068.

Lancaster, M.A., and Knoblich, J.A. (2014). Generation of cerebral organoids from human pluripotent stem cells. Nat Protoc *9*, 2329-2340.

Li, H.L., Gee, P., Ishida, K., and Hotta, A. (2016). Efficient genomic correction methods in human iPS cells using CRISPR-Cas9 system. Methods *101*, 27-35.

Nagashima, F., Suzuki, I.K., Shitamukai, A., Sakaguchi, H., Iwashita, M., Kobayashi, T., Tone, S., Toida, K., Vanderhaeghen, P., and Kosodo, Y. (2014). Novel and robust transplantation reveals the acquisition of polarized processes by cortical cells derived from mouse and human pluripotent stem cells. Stem Cells Dev *23*, 2129-2142.

Oceguera-Yanez, F., Kim, S.I., Matsumoto, T., Tan, G.W., Xiang, L., Hatani, T., Kondo, T., Ikeya, M., Yoshida, Y., Inoue, H.*, et al.* (2016). Engineering the AAVS1 locus for consistent and scalable transgene expression in human iPSCs and their differentiated derivatives. Methods *101*, 43-55.

Shitamukai, A., Konno, D., and Matsuzaki, F. (2011). Oblique radial glial divisions in the developing mouse neocortex induce self-renewing progenitors outside the germinal zone that resemble primate outer subventricular zone progenitors. J Neurosci *31*, 3683-3695.

Susaki, E.A., Tainaka, K., Perrin, D., Kishino, F., Tawara, T., Watanabe, T.M., Yokoyama, C., Onoe, H., Eguchi, M., Yamaguchi, S.*, et al.* (2014). Whole-brain imaging with single-cell resolution using chemical cocktails and computational analysis. Cell *157*, 726-739.
