# Supplementary material for: Direct visualization of the transition status during neural differentiation by dual-fluorescent reporter human pluripotent stem cells
Source: Stem Cell Reports. 2022 Aug 4;17(9):1903–13. doi: 10.1016/j.stemcr.2022.07.001 (PMC9481873; doi:10.1016/j.stemcr.2022.07.001)
Supplement: Document S2. Article plus supplemental information [file mmc4.pdf]

Stem Cell Reports
Report
OPEN ACCESS

Direct visualization of the transition status during neural differentiation by dual-fluorescent reporter human pluripotent stem cells

Gwanghyun Park,[1] Minkyung Shin,[1,4] Wonyoung Lee,[1,4] Akitsu Hotta,[2] Taeko Kobayashi,[3] and Yoichi Kosodo[1,*]
[1]Korea Brain Research Institute (KBRI), Daegu 41068, Republic of Korea
[2]Center for iPS Cell Research and Application (CiRA), Kyoto University, Kyoto 606-8507, Japan
[3]Research Center for Dynamic Living Systems, Graduate School of Biostudies, Kyoto University, Kyoto 606-8501, Japan
[4]These authors contributed equally
*Correspondence: kosodo@kbri.re.kr
https://doi.org/10.1016/j.stemcr.2022.07.001

SUMMARY

Human induced pluripotent stem cells (hiPSCs) can differentiate into neurons and glia via neural progenitor cells and are widely used for neurogenic studies. However, directly visualizing the transition status during the neural differentiation of live cells is difficult. Here, targeting NEUROG2 (NGN2) and TUBB3 as markers of neurogenic cells and neurons, respectively, we established TUBB3^{EGFP}/NGN2^{TagRFP} dual-reporter hiPSCs using CRISPR-Cas9 technology. We induced the differentiation of cortical neurons from dual-reporter hiPSCs, successfully visualizing cell-fate conversion in two-dimensional (2D) culture and 3D organoids. The reporter cells were used to monitor drug effects to enhance neural induction, responses to gene knockdown, transplantation to the embryonic mouse brain, and live imaging at single-cell resolution. Notably, the earliest REELIN-positive neurons showed a distinctive migration pattern, and their production was accelerated by HES1-function loss. Together, these results demonstrate the potential for dual-reporter hiPSCs in therapeutic neural regeneration strategies and studies on human cortical development.

INTRODUCTION

Human induced pluripotent stem cells (hiPSCs) have many advantages for modeling development and disease, cell therapy and drug screening. Numerous studies have reported the production of neural and glial cells in the central nervous system (CNS) via neural progenitor cells from hiPSCs and human embryonic stem cells (ESCs). Considering the use of such cells for therapeutic purposes such as drug testing and transplantation in animal models, monitoring the differentiation status of living cells would provide vital information. To do this, the introduction of fluorescent reporter protein coding sequences into the loci of specific marker genes by genome-editing technology has shown promise (Hotta and Yamanaka, 2015).

To date, several fluorescent reporter cell lines that distinguish the neural differentiation status have been established, such as NEUROG2 (NGN2) for neurogenic cells (Li et al., 2015) and tyrosine hydroxylase for dopaminergic neurons (Calatayud et al., 2019). Although these “single” fluorescent reporter cells can distinguish differentiated cells from pluripotent stem cells, differentiation into somatic cells generally requires many steps, and several intermediate cell populations appear prior to terminal differentiation. To trace multiple differentiation statuses in live cells, a dual-fluorescent reporter system has been shown to be useful for some cellular lineages. Various cardiac cells derived from TBX5^{Clover2}/NKX2-5^{TagRFP} hiPSCs were used for precise drug testing (Zhang et al., 2019). Likewise, PAX7^{tdTomato}/MYF5^{EGFP} and NKX2-1^{EGFP}/TP63^{tdTomato} reporter cells allowed the successful characterization of skeletal muscle progenitors (Wu et al., 2018) and airway basal stem cells (Hawkins et al., 2021), respectively.

Regarding the cortical neural lineage, neuroepithelial stem cells typically differentiate into radial glial cells and then into intermediate progenitors. Such cells are categorized as neural progenitors because of their ability to produce postmitotic neurons. Intriguingly, a specific subtype of neural progenitors named “outer glial cells” are abundant in mammals within the gyrified cortex and may play critical roles in neocortical expansion (Kalebic and Huttner, 2020). Therefore, monitoring transient status during neural differentiation with multiple reporters would become a strong and versatile approach not only for therapeutic strategies, such as screening and efficient production of specific neurons, but also for elucidating developmental programs, especially in humans.

In this study, using NGN2 and Tubulin β-III (TUBB3) as markers of neurogenic cells and neurons, respectively, we established TUBB3^{EGFP}/NGN2^{TagRFP} dual-color fluorescent reporter hiPSCs followed by characterization using two-dimensional (2D) and 3D culture systems. The dual-reporter cells were used to monitor drug responses, gene knockdown (KD), and transplantation to embryonic mouse brains. We further performed live imaging to assess migration characteristics. By combining these approaches, we discovered that the appearance of the earliest neurons in the human neural lineage was accelerated by the KD of

© 2022 The Author(s). This is an open access article under the CC BY license (http://creativecommons.org/licenses/by/4.0/).

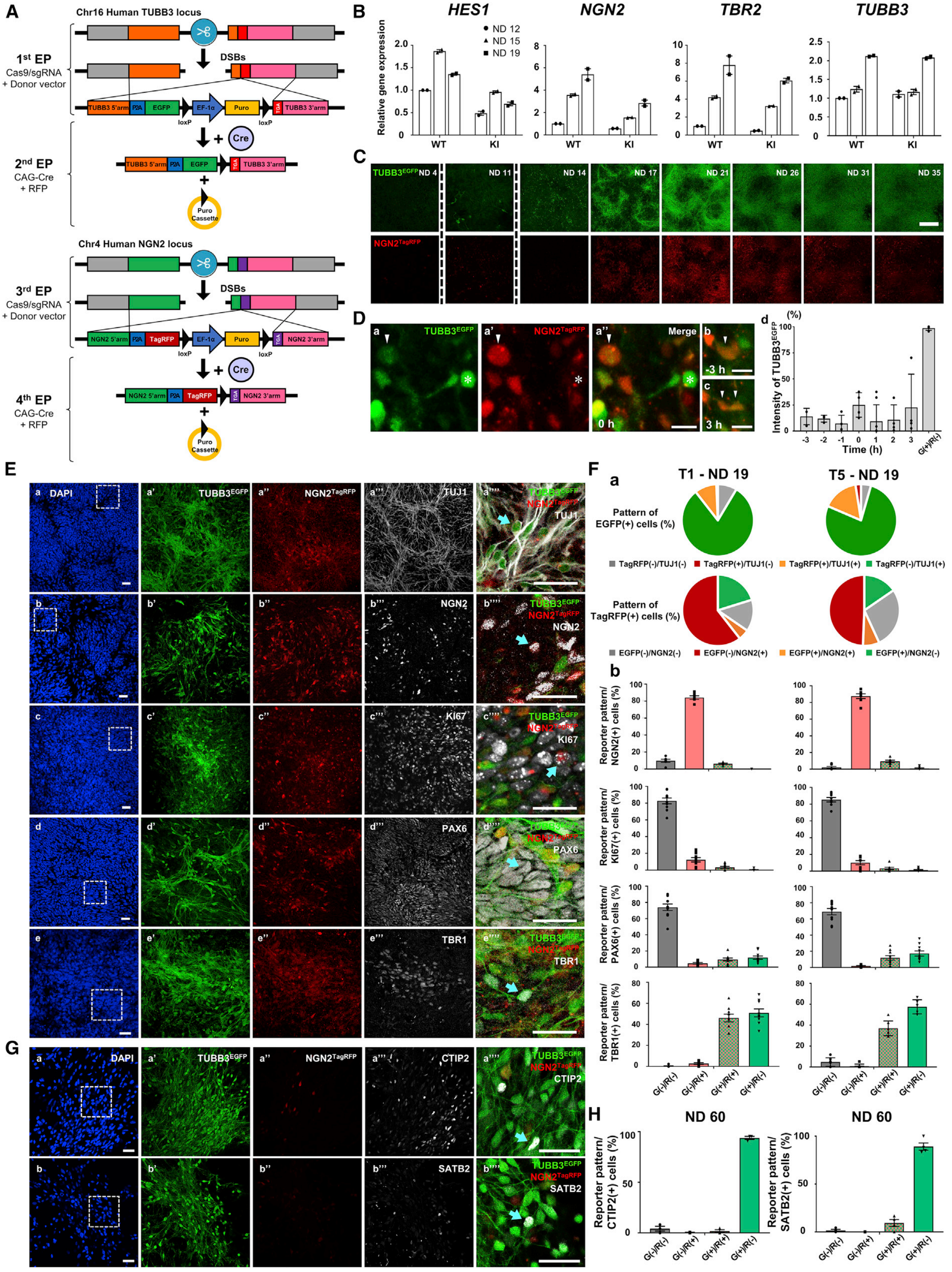

(legend on next page)

HES1, a basic helix-loop-helix transcription factor. Furthermore, the earliest induced neurons were enriched for the REELIN (RELN)-positive neural subtype. These results demonstrate the usefulness of dual-color reporters to visualize shifts in human neural lineage in live cells for a number of applications.

RESULTS AND DISCUSSION

Establishment and characterization of TUBB3^{EGFP}/NGN2^{TagRFP} dual-reporter hiPSCs

To generate dual-reporter hiPSCs, multi-step electroporation (EP) of CRISPR-Cas9 genome editing and Cre-recombination were performed (Figure 1A). Single-guide RNAs (sgRNAs) and homology arms were designed to add the coding sequences of EGFP and TagRFP with the self-cleaving peptide P2A (Kim et al., 2011) near the stop codons of TUBB3 and NGN2, respectively (Figures 1A and S1). For the host hiPSCs, the RNA-based reprogrammed RPChiPS771 line, which has sufficient ability to differentiate into three germ layers and produce cortical neurons (Iwashita et al., 2019), was selected among 4 hiPSC lines. We selected TUBB3^{EGFP}/NGN2^{TagRFP} heterozygous knockin (KI) clones (Figure S1) after confirming their normal karyotype and pluripotency (Figure S1) in lines with different sgRNA target sites for NGN2 (named T1 and T5). Subsequently, we induced the differentiation of neural cells into cortical cells using the 2D culture method (Espuny-Camacho et al., 2013) and assessed whether the genome modifications influenced neural differentiation by examining the transcript levels of marker genes (*HES1*, *NGN2*, *TBR2*, and *TUBB3*). The neural induction profiles of cells derived from the dual-reporter and parental hiPSCs showed similar fluctuation patterns along 12, 15, and 19 days (Figure 1B). We then monitored the expression of fluorescent proteins during neural differentiation for 5 weeks (Figures 1C and S2). In both the T1 and T5 lines, distinct expression patterns of EGFP and TagRFP were observed as early as neural differentiation day (ND) 11, whereas these signals were not visible in the initial stage (ND 4). Notably, the EGFP signal increased and was maintained over time, while the TagRFP signal peaked at approximately ND 20–24 and thereafter became weaker (Figures 1C and S2). Time-lapse recordings allowed us to identify dividing NGN2^{TagRFP}-positive (R^+) cells (Figure 1D; Video S1). Although a faint signal of EGFP was detected during mitosis, the intensity level was far less than TUBB3^{EGFP}-positive (G^+) cells without a TagRFP signal. These results indicated that R^+ cells appeared as neurogenic cells and then were consumed to produce neurons, while G^+ cells remained as postmitotic neurons.

To perform further analyses at the cellular resolution, immunostaining was used to evaluate neural cells at ND 19, the time point at which proliferative cells, progenitor cells, and postmitotic neurons exist simultaneously (Figures 1E and 1F). We first compared endogenous TUBB3 and NGN2 proteins with EGFP and TagRFP fluorescent reporters, respectively. The vast majority of G^+ cells overlapped with the antibody for TUBB3 (TUJ1) staining (91% [T1] and 94% [T5]). In the case of R^+ cells, overlapped cells with the antibody for NGN2 were 65% (T1) and 57% (T5), and 20% (T1) and 15% (T5) were G^+/NGN2$^-$. Among NGN2$^+$ cells, 84% (T1) and 88% (T5) were R^+. A part of KI67-, a proliferative marker, positive cells were R^+ (12% [T1] and 10% [T5]). TBR1, a marker of newborn cortical neurons, overlapped not only with the G^+ populations but also with G^+/R^+ cells (46% [T1] and 37% [T5]). These results suggest that TagRFP protein can remain temporally after endogenous NGN2 protein is diminished in newborn

Figure 1. Establishment and characterization of dual-reporter hiPSC-derived neural cells

(A) Schematic of EP to establish dual-reporter hiPSC lines.

(B) qRT-PCR analysis of the original (wild-type [WT]) and dual-reporter hiPSC (T1)-derived neural cells (KI) on NDs 12, 15, and 19. The expression level for each gene was normalized to that on ND 12 from WT. Independent experiments (IEs), 2.

(C) Sequential images during neural differentiation (T1, up to ND 35) at the same position on the grid culture dish. Note that images at unspecified positions are presented for NDs 4 and 11 because of faint signals at the stages. Bar: 300 μm.

(D) (a–c) Division of R^+ cell (arrowheads) (NDs 14–15, T1). Time point 0 corresponds to the time just before the separation of daughter cells. Bars: 15 μm. (d) EGFP signal intensity of 4 dividing cells (5 IEs). A G^+/R^- cell (asterisks) was used as the reference (100%) among recorded images.

(E) Immunostaining images obtained using TUJ1 (a), NGN2 (b), KI67 (c), PAX6 (d), and TBR1 (e) antibodies fixed on ND 19. (a″″)–(e″″) show magnified views of the dashed squares in (a)–(e), respectively. The arrows indicate TUJ1$^+$/G^+ (a″″), NGN2$^+$/R^+ (b″″), KI67$^+$/R^+ (c″″), PAX6$^+$ and G^-/R^- (d″″), and TBR1$^+$/G^+ cells (e″″). Bar: 30 μm.

(F) (a) (Top) Populations of R^+ and TUJ1$^+$ among G^+ cells. (Bottom) Populations of G^+ and NGN2$^+$ among R^+ cells. (b) Quantification of G^+ and R^+ among the NGN2$^+$, PAX6$^+$, TBR1$^+$, and KI67$^+$ cells in (E). Number of fields, 6–10 for each T1 and T5 from 3 IEs.

(G) Immunostaining images obtained using CTIP2 (a) and SATB2 (b) antibodies after long-term neural differentiation (T5, ND 60). (a″″) and (b″″) show magnified views of the dashed squares in (a) and (b), respectively. The arrows indicate CTIP2$^+$/G^+ (a″″) and SATB2$^+$/G^+ cells (b″″). Bar: 30 μm.

(H) Quantification of G^+ and R^+ among the CTIP2$^+$ and SATB2$^+$ cells in (G). Number of fields, 3–4 from 2 IEs.

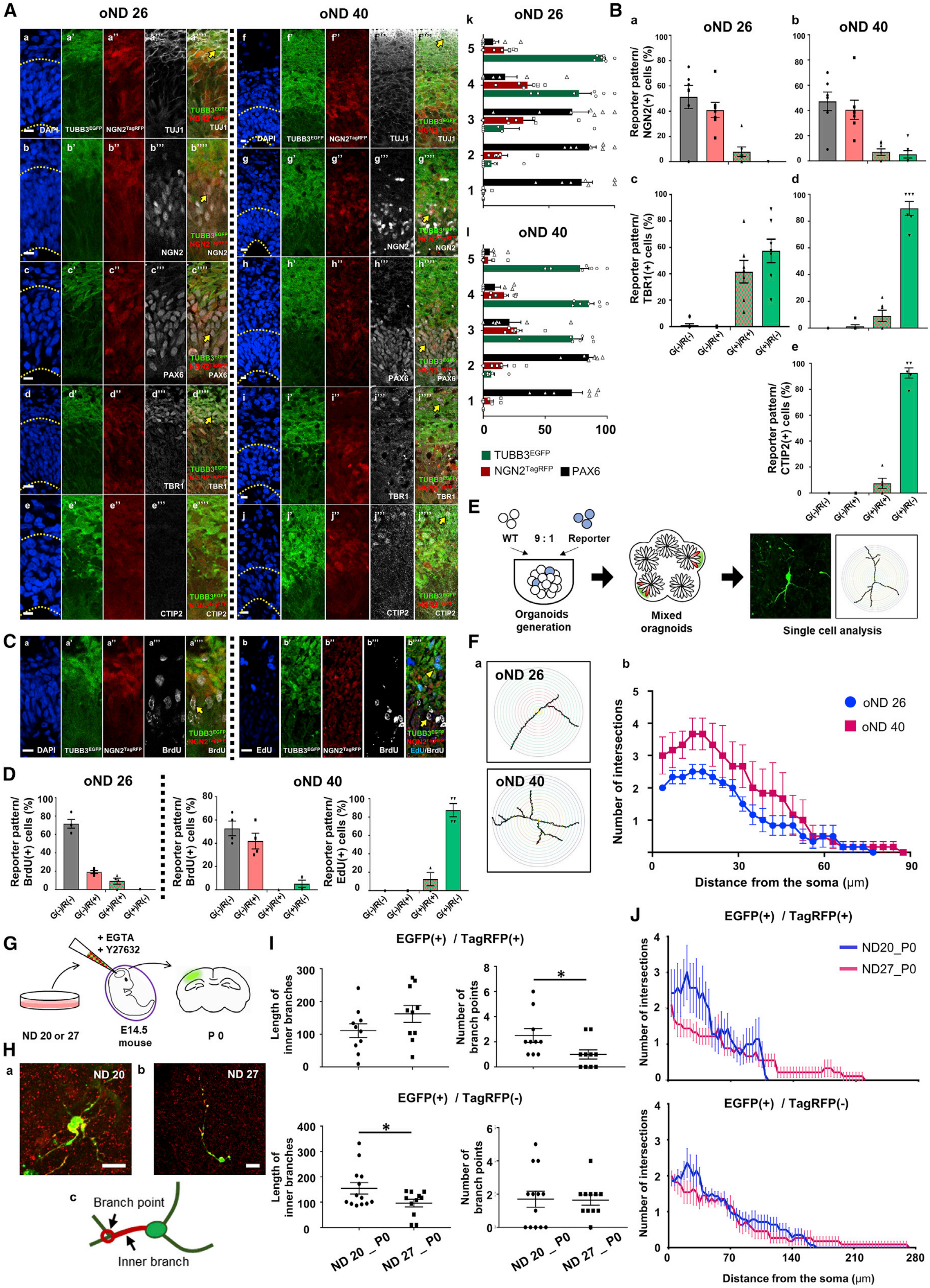

(legend on next page)

neurons. In future studies, use of destabilized RFP variants might improve the temporal sensitivity. We further assessed long-term neural differentiation (ND 60). Among CTIP2^{+} (marker of layer 5 neurons) and SATB2^{+} (marker of layer 2/3 neurons) cells, 94% and 89% were G^{+}, respectively, indicating that EGFP continued to be expressed in terminally differentiated neurons (Figures 1G and 1H). Taken together, TUBB3^{EGFP}/NGN2^{TagRFP} dual-reporter hiPSCs can differentiate into cortical neurons, and the expression of each fluorescent reporter reflects neural fate conversion.

Cerebral organoid generation and xenograft transplantation using dual-reporter cells

We attempted to characterize the dual-reporter cells in a 3D environment in applications involving cerebral organoids and xenograft transplantation to the developing mouse brain. We cultured organoids for 26 and 40 days (oNDs 26 and 40) after the initiation of embryoid body (EB) formation and then evaluated the expression of fluorescent reporters and cell positions both inside and outside the ventricular zone (VZ), as defined by PAX6, a neural stem cell (NSC) marker, immunostaining (Figures 2A and S3). G^{+} cells were localized peripherally in the VZ, while R^{+} cells were observed between the G^{+} layer and the VZ. Furthermore, at oND 40, the VZ was thinner, while the TUJ1 immunopositive G^{+}-layer was thicker than that on oND 26 (Figure 2A). These results indicated that TagRFP and EGFP were sequentially expressed while cells were migrating out from the VZ. Among NGN2^{+} cells at both oNDs 26 and 40, approximately 40% and 7%–8% were R^{+} and G^{+}/R^{+}, respectively, while 50% of cells were R^{-}, mostly localizing at the apical region of the VZ (Figures 2A and 2B). This staining feature resembled the pattern of the Neurogenin2 fluorescent reporter in the developing cortices of *Ngn2*-EGFP Tg mice (Figure S4). We further evaluated the expression of TBR1 and CTIP2 to examine neural subtypes in the organoids (Figures 2A and 2B). Around half of TBR1^{+} cells were G^{+}/R^{+} at oND 26, while most of TBR1^{+} cells were G^{+}/R^{-} at oND 40. The results suggest that the generation of TBR1^{+} neurons is intensive at oND 26 and decreases at oND 40. This is because the TagRFP remaining in G^{+} cells should represent newly born neurons, as observed in 2D culture. CTIP2^{+} cells were rarely observed at oND 26 while appearing at oND 40, which reflects the chronologicity of corticogenesis.

We next analyzed the mitotic activity of R^{+} cells and the migration of G^{+} cells in the organoids using EdU and BrdU, thymidine analogs incorporated by proliferative cells during the S phase. First, we treated oND 26 cells with BrdU prior to fixation and observed that BrdU^{+} cells were sparsely distributed in the VZ (Figure 2C). Importantly, 19% of BrdU^{+} cells were R^{+}, indicating that they expressed NGN2^{TagRFP} during or immediately after the S phase. Next, EdU was supplied for 4 h on oND 26, and BrdU was administered on oND 40. Most EdU^{+} cells were G^{+} (88%), while 42% of BrdU^{+} cells were R^{+} (Figure 2D). These results confirm that R^{+} cells have the ability of mitosis in the neurogenic niche in organoids as well as in 2D culture, while G^{+} cells migrate to the basal side as postmitotic neurons.

We performed quantitative morphological assays at the single-cell level by culturing heterogeneous cell populations to form "mosaic" organoids (Figure 2E). Dual-reporter and parental hiPSCs were mixed at a ratio of 1 to 9 at the EB-formation step. Sholl analysis revealed

Figure 2. Analysis of dual-reporter hiPSC-derived neural cells in brain organoids and the developing mouse brain

(A) Immunostaining images obtained using TUJ1 (a and f), NGN2 (b and g), PAX6 (c and h), TBR1 (d and i), and CTIP2 (e and j) antibodies fixed on oNDs 26 and 40 (T1). The arrows indicate TUJ1^{+}/G^{+} (a'''' and f''''), NGN2^{+}/R^{+} (b'''' and g''''), PAX6^{+} and G^{-}/R^{-} (c'''' and h''''), TBR1^{+}/G^{+} (d'''' and i''''), and CTIP2^{+}/G^{+} (j'''') cells. Dotted line (a–j): border of VZ. Bar: 10 μm. (k and l) Ratios of G^{+}, R^{+}, and PAX6^{+} cells at oNDs 26 (c) and 40 (h) (%, per DAPI). The cortical structure from apical to basal surface is evenly divided into bins 1–5. Number of fields, 7 from 7 organoids (3 IEs).

(B) Quantification of G^{+} and R^{+} among the NGN2^{+}, TBR1^{+}, and CTIP2^{+} cells in (A). Number of fields, 7 from 7 organoids (3 IEs).

(C) BrdU/EdU labeling to assess the proliferation and migration (T5). (a) BrdU was applied 4 h before fixation on oND 26. (b) EdU and BrdU were applied for 14 days and 4 h, respectively, before fixation on oND 40. The arrows and arrowheads indicate BrdU^{+}/R^{+} (a'''' and b'''') and EdU^{+}/G^{+} (b'''') cells, respectively. Bar: 10 μm.

(D) Quantification of the images in (C). Number of fields, 4 from 4 organoids (2 IEs).

(E) Schematic of mixed organoid generation using WT and dual-reporter hiPSCs.

(F) Representative tracing images of G^{+} cells as analyzed by the Sholl method in mixed organoids (a) and quantification (b) (6 T1 cells at both oNDs 26 and 40 from 2 IEs).

(G) Schematic of the transplantation of dual-reporter cells into the lateral ventricles of embryonic mice.

(H) Representative images of G^{+}/R^{+} cells using ND 20 (a) and 27 (b) donor cells (T1). (c) Parameters for morphological quantification. Bar: 20 μm.

(I) Quantification of the parameters indicated in (H) for G^{+}/R^{+} (n = 10 for NDs 20 and 27) and G^{+} cells (n = 13 and 11 for NDs 20 and 27, respectively) (3 IEs).

(J) Quantification of the number of intersections of grafted dual-reporter cells (same sample as I).

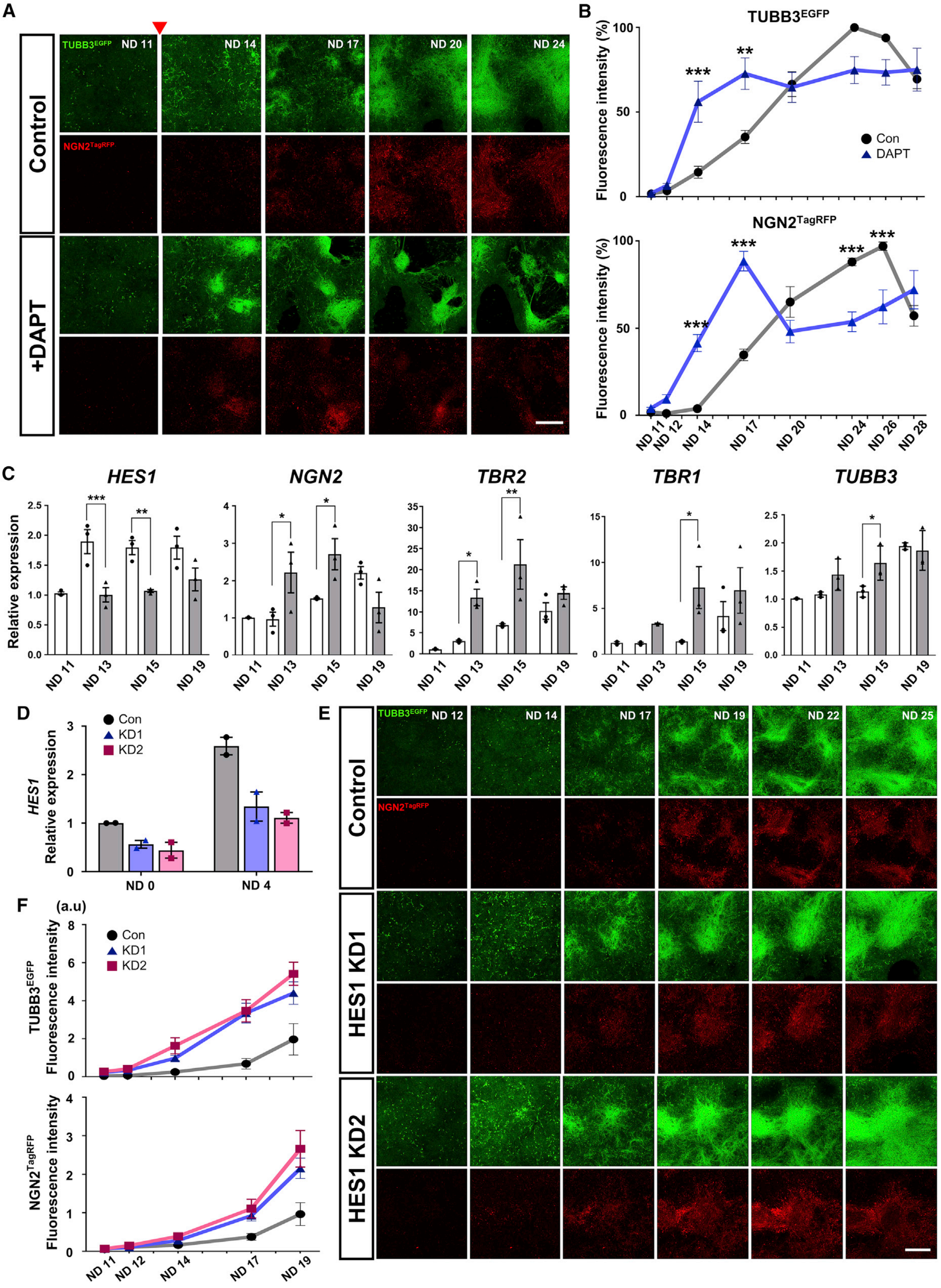

(legend on next page)

that G^+ cells had fewer intersections close to the soma at oND 26 compared with at oND 40 (Figure 2F), indicating an increase of neurites in organoids over time.

The morphology of dual-reporter cells at the single-cell level was further examined in the developing mouse cortex using an efficient transplantation method that we previously established (Nagashima et al., 2014) (Figure 2G). Dual-reporter cells were transplanted into the embryonic day 14.5 (E14.5) brain on NDs 20 and 27, followed by dissection at birth. Due to the small number of R^+ cells, we compared the morphological parameters of the G^+/R^+ and G^+ populations (Figure 2H). More branch points and intersections were observed in the G^+/R^+ population transplanted on ND 20 than on those transplanted on ND 27, a feature not observed in the G^+ population (Figures 2I and 2J). These results indicated that the number of branched shapes in newborn neurons were directly associated with the period of neural induction, with more branched shapes being correlated with a shorter period of induction. Accordingly, dual-reporter cells allow complex morphological analyses of specific cell types after xenograft transplantation.

Application of dual-reporter cells for drug response and gene function analyses

Reporter cells can be used to monitor the differentiation status of cells in real time during culture. We next assessed the applicability of dual-reporter cells for this purpose by evaluating the Notch signaling pathway, a key regulator of NSC proliferation and differentiation (Kobayashi and Kageyama, 2011). We used dual-reporter cells to visualize neural lineage conversion via the Notch signal inhibitor DAPT, a gamma-secretase inhibitor (Crawford and Roelink, 2007). DAPT was administered on ND 11, and the expression of fluorescent reporters was monitored by sequential imaging. On NDs 14–17, the numbers of both G^+ and R^+ cells were significantly increased by DAPT treatment (Figures 3A and 3B). To confirm the potentiated neurogenesis, qRT-PCR was performed to monitor neural differentiation markers over time. High expression levels of *NGN2*, *TBR2*, *TUBB3*, and *TBR1* were observed in the DAPT-treated samples on NDs 13 and 15. In contrast, the level of *HES1*, a pivotal mediator of the Notch pathway, was decreased during the same period (Figure 3C).

Based on these results, we further attempted to monitor the neural differentiation of live cells after manipulating the function of the HES1 gene. A previous report demonstrated that the loss of Hes1 function in mouse ESCs strongly enhanced neural production (Kobayashi et al., 2009). Here, we infected undifferentiated dual-reporter cells with a lentivirus encoding HES1 short hairpin RNA (shRNA) (Figure S4). After confirming HES1 KD at the early ND stage (Figure 3D), we monitored fluorescent protein expression during neural induction. Both the G^+ and R^+ populations appeared earlier after HES1 KD based on an increased fluorescence intensity, indicating that HES1 KD accelerates neural differentiation (Figures 3E and 3F). A reduction in the level of HES1 may preferentially lead to neural lineages, possibly due to the loss of heterogeneity in both the differentiation timing and fate choice (Kobayashi and Kageyama, 2011). These results demonstrate that neural fate conversions induced by drug and gene modulation were validated in real time and that dual-reporter cells can be utilized for screening strategies.

Tracking cell-migration patterns during neural differentiation

Intensive migration activity is a key feature of neural cells. Although numerous studies on neural migration have been performed using animal models, the migration mechanisms of human neural cells must be elucidated to further understand the expanded cerebral cortex in humans. Here, we characterized the migration pattern of reporter-expressing cells by time-lapse recording followed by quantitative tracking of fluorescent signals. Occasionally, we observed a transition from R^+ to G^+ cells at the single-cell level (Figure 4A; Video S2). We compared the characteristics of migration using track straightness (TS) and track speed max (TSM) parameters, defined by the degree of monodirectional migration and the highest speed during the recording period, respectively. We found that G^+ exhibited a higher TS value than R^+ cells from ND 12 to 13 (Figures 4B and 4C). This result suggests that the migration mode of

Figure 3. Monitoring the temporal potentiation of neural differentiation using drug treatment and gene KD strategies

(A) Sequential images of G^+ and R^+ cells (T1) at the same position on the culture dish after treatment with DAPT (5 μM) starting on ND 11 (arrowhead). Bar: 300 μm.

(B) Quantification of the fluorescence intensities of the images in (A). Gray for the control and blue for the DAPT-treated cells. Number of fields, 9–10 from 3 IEs.

(C) qRT-PCR analysis of neural marker genes in the control (white) and DAPT (gray)-treated cells. The expression levels were normalized by those in the control group on ND 11 (3 IEs).

(D) qRT-PCR analysis of HES1 KD dual-reporter cells (T1) on NDs 0 (iPSC stage) and 4. The expression levels were normalized to those in the control group on ND 0. Two different shRNA constructs (KD1 and 2) were utilized (2 IEs).

(E) Sequential images of G^+ and R^+ cells (T1) with HES1 KD at the same position on the culture dish. Bar: 300 μm.

(F) Quantification of the fluorescence intensities (arbitrary unit) of the images in (E). Number of fields, 7 from 2 IEs.

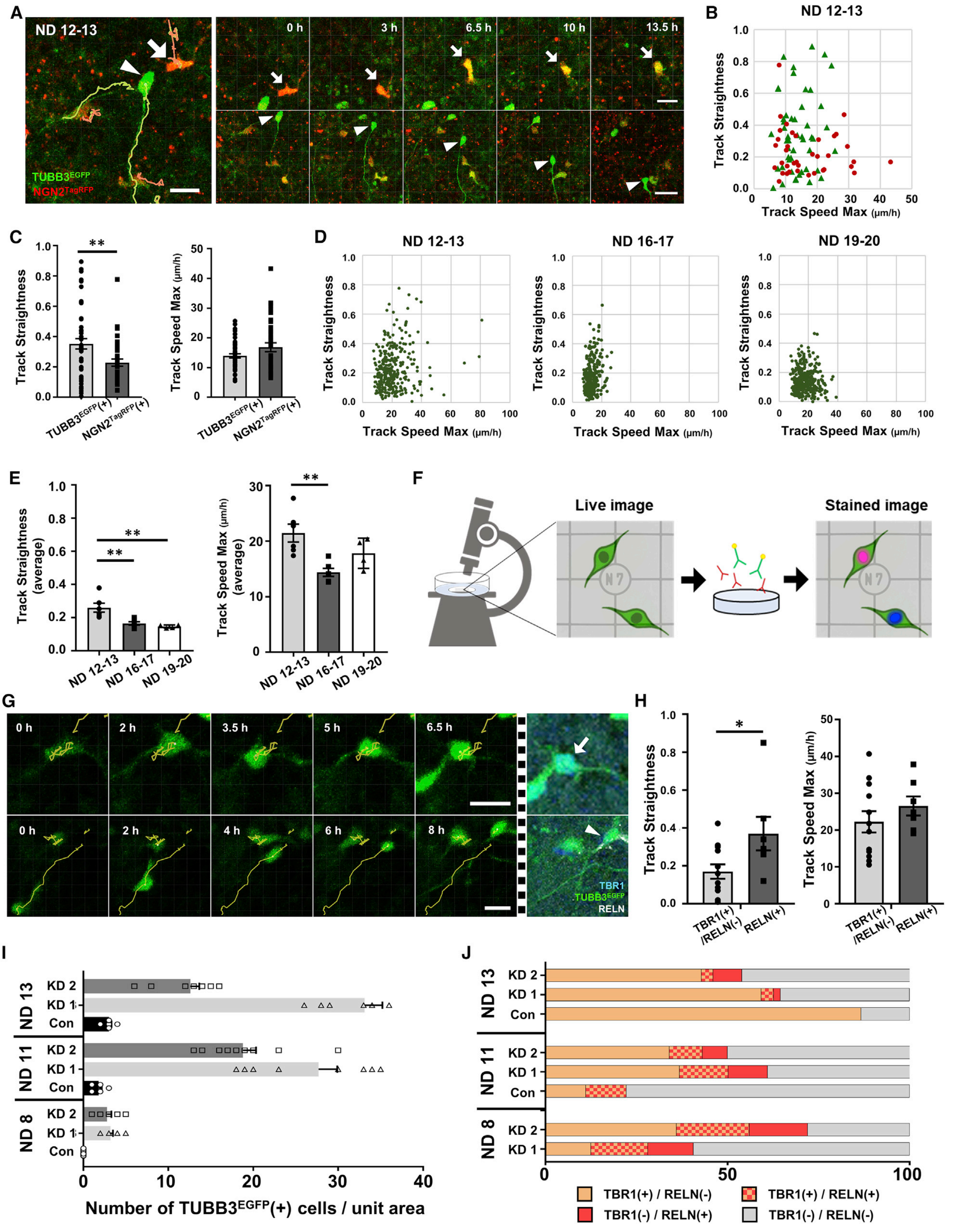

(legend on next page)

human neurons is altered after exit from the neurogenic state, as observed in the embryonic mouse cortex (Tabata and Nakajima, 2003).

To examine the migration ability of neurons depending on the neural induction period, we compared numerous tracking records of G^+ cells starting on NDs 12, 16, and 19 (Figures 4D and 4E). Importantly, distinct populations had higher TS and TSM values from NDs 12 to 13 than at the later stages. This finding prompted us to further elucidate migration abilities among early-born neurons. During human cortical development, RELN-positive Cajal-Retzius (CR) cells appear as early as 5 gestational weeks (Meyer et al., 2000). The subpopulation of CR cells originating from the pallium is double positive for RELN and TBR1 (Hevner et al., 2003; Meyer and Gonzalez-Gomez, 2018). To investigate the correlation between neural migration pattern and subtype among early-born neurons, we conducted cellular tracking followed by immunostaining with RELN and TBR1 antibodies (Figure 4F). Notably, the migration modes of TBR1 single- and RELN-positive G^+ cells were compared, and the latter had a higher TS (Figures 4G and 4H), possibly explaining the phenomenon of long-distance migration during cortical formation.

Next, we explored whether the proportion of $RELN^+$ cells was altered in ectopic neurons induced by HES1 KD. We found that the earliest G^+ cells appeared at ND 8 after HES1 KD, while essentially no population appeared in the control group (Figure 4I). Strikingly, $RELN^+$ cells accounted for 28% and 36% of HES1 KD1 and KD2 cells, respectively, at ND 8 (Figure 4J). Among them, approximately half of the populations were double positive for TBR1 and RELN, a feature of pallium-derived CR cells. Together, the dual-reporter cells enabled the characterization of the migration mode of early-born neural cells in correlation with their subtypes and revealed the role of HES1 in the efficient production of RELN-positive neurons in the human neural lineage.

CR cell numbers are reported to be increased in the embryonic mouse brain during the early neurogenic stage upon Hes1/3/5 triple knockout (Imayoshi et al., 2008), suggesting the existence of a common mechanism for the production of RELN-positive neurons regulated by the Hes family in the mammalian neural lineage. Clinically, RELN is known to have several neural functions, and its loss may induce brain disorders. Notably, CR cells serve as the main source of Reelin in the developing brain, while Reelin in the adult brain is produced by a subpopulation of GABAergic interneurons, indicating that Reelin may have differential functions in developing and adult brains (Ishii et al., 2016). In the developing brain, Reelin plays important roles in not only the formation of cortical layers, as shown by classical studies on reeler mice, but also neural migration and signaling pathways (Santana and Marzolo, 2017). In addition, the mutation of RELN can cause neuropsychiatric disorders, such as schizophrenia and autism. Here, HES1 KD significantly increased the proportion of RELN-positive neurons. The induction method can be assessed using patient-derived hiPSCs to characterize RELN dysfunction both *in vitro* and *in vivo* after transplantation in model animals.

EXPERIMENTAL PROCEDURES

hiPSC lines

Use of human-derived material in the research is approved by the Research Ethics Committee of KBRI (KBRI-201603-BR-001-01). The hiPSC line used in this study (RPChiPS771, ReproCELL, Yokohama, Japan) was characterized in a previous publication (Iwashita et al., 2019). Briefly, hiPSCs were obtained by the self-replicative RNA reprogramming method. Pluripotency toward three germ layers and higher efficiency toward neural lineage compared with other established hiPSC lines (1231A3, 1383D2, and 1383D6, CiRA, Kyoto University) were confirmed by qRT-PCR.

Statistical analysis

Prism (GraphPad Software) was used for statistical analyses. Differences between two groups were analyzed by the two-tailed Student's t test, and differences between more than two groups

Figure 4. Cell-migration analysis of neural differentiation in correlation with RELN expression

(A) Sequential images of cell-fate transition and neural migration (NDs 12–13, T1). Note that R^+ occasionally converted into G^+/R^+ cells during the recording (arrow). G^+ cells tended to show monodirectional migration (arrowhead). Bar: 20 μm.

(B) Quantification of the TS and TSM values for G^+ (triangle) and R^+ (circle) cells (NDs 12–13, T1, merge of 3 IEs).

(C) Statistic analysis of the data in (B).

(D) Differential migration patterns of G^+ cells from NDs 12–13, 16–17, and 19–20. Approximately 300 tracked cells from 5 IEs at each stage are presented.

(E) Statistic analysis of the data in (D).

(F) Schematic of subtype specification by immunostaining after live imaging.

(G) Sequential tracking images of migrating G^+ cells that were $TBR1^+/RELN^-$ (arrow) and $RELN^+/TBR1^-$ (arrowhead). The dashed bar indicates the time point of fixation. Bar: 20 μm.

(H) Statistical analysis of migration parameters for $TBR1^+/RELN^-$ and $RELN^+$ cells (NDs 12–13, T1, 3 IEs).

(I) Number of G^+ cells per unit area on NDs 8, 11, and 13 after HES1 KD (T1, 3 IEs).

(J) Ratios of $TBR1^+$ and $RELN^+$ cells after HES1 KD in (I).

were analyzed by ANOVA followed by Tukey's test. Differences were considered significant for p values <0.05 (*$p < 0.05$, **$p < 0.01$, and ***$p < 0.001$). All error bars represent the SEM. For qRT-PCR analysis, each point indicates the averaged value of triplicated reactions.

Other materials and methods

All animal protocols were approved by the KBRI institutional animal care and use committee (IACUC-20-00044). Other materials and methods are described in the supplemental experimental procedures.

SUPPLEMENTAL INFORMATION

Supplemental information can be found online at https://doi.org/10.1016/j.stemcr.2022.07.001.

AUTHOR CONTRIBUTIONS

Study design and conceptualization, G.P. and Y.K.; manuscript preparation, G.P. and Y.K.; experiments, G.P. for all, M.S. for imaging of cell divisions, and W.L. for transplantation; resources, A.H. and T.K.; project administration, Y.K.

ACKNOWLEDGMENTS

The authors thank Carina Hanashima and Ikuo Suzuki for critically reading the manuscript; Makoto Ikeya, Mineko Kengaku, Misato Iwashita, and Youngjae Ryu for valuable support and discussion; the KBRI Research Equipment Core Facility Team and Fumiyoshi Ishidate for technical assistance; and Karan Ishii for text editing. This study was funded by grants from the KBRI Basic Research Program of the Ministry of Science, ICT, and Future Planning (22-BR-02-01, 22-BR-03-01 to Y.K.), the National Research Foundation of Korea (2022R1A2C1002728 to Y.K.), the Japan Agency for Medical Research and Development (JP20gm6410006 to T.K.), and SPIRITS 2021 of Kyoto University (to T.K.).

CONFLICTS OF INTERESTS

The authors have no conflicts of interest to declare.

Received: January 10, 2022
Revised: June 30, 2022
Accepted: July 1, 2022
Published: August 4, 2022

REFERENCES

Calatayud, C., Carola, G., Fernández-Carasa, I., Valtorta, M., Jiménez-Delgado, S., Díaz, M., Soriano-Fradera, J., Cappelletti, G., García-Sancho, J., Raya, Á., et al. (2019). CRISPR/Cas9-mediated generation of a tyrosine hydroxylase reporter iPSC line for live imaging and isolation of dopaminergic neurons. Sci. Rep. *9*, 6811.

Crawford, T.Q., and Roelink, H. (2007). The Notch response inhibitor DAPT enhances neuronal differentiation in embryonic stem cell-derived embryoid bodies independently of Sonic Hedgehog signaling. Dev. Dyn. *236*, 886–892.

Espuny-Camacho, I., Michelsen, K.A., Gall, D., Linaro, D., Hasche, A., Bonnefont, J., Bali, C., Orduz, D., Bilheu, A., Herpoel, A., et al. (2013). Pyramidal neurons derived from human pluripotent stem cells integrate efficiently into mouse brain circuits *in vivo*. Neuron *77*, 440–456.

Hawkins, F.J., Suzuki, S., Beermann, M.L., Barillà, C., Wang, R., Villacorta-Martin, C., Berical, A., Jean, J.C., Le Suer, J., Matte, T., et al. (2021). Derivation of airway basal stem cells from human pluripotent stem cells. Cell Stem Cell *28*, 79–95.e8.

Hevner, R.F., Neogi, T., Englund, C., Daza, R.A.M., and Fink, A. (2003). Cajal-Retzius cells in the mouse: transcription factors, neurotransmitters, and birthdays suggest a pallial origin. Brain Res. Dev. Brain Res. *141*, 39–53.

Hotta, A., and Yamanaka, S. (2015). From genomics to gene therapy: induced pluripotent stem cells meet genome editing. Annu. Rev. Genet. *49*, 47–70.

Imayoshi, I., Shimogori, T., Ohtsuka, T., and Kageyama, R. (2008). Hes genes and neurogenin regulate non-neural versus neural fate specification in the dorsal telencephalic midline. Development *135*, 2531–2541.

Ishii, K., Kubo, K.I., and Nakajima, K. (2016). Reelin and neuropsychiatric disorders. Front. Cell. Neurosci. *10*, 229.

Iwashita, M., Ohta, H., Fujisawa, T., Cho, M., Ikeya, M., Kidoaki, S., and Kosodo, Y. (2019). Brain-stiffness-mimicking tilapia collagen gel promotes the induction of dorsal cortical neurons from human pluripotent stem cells. Sci. Rep. *9*, 3068.

Kalebic, N., and Huttner, W.B. (2020). Basal progenitor morphology and neocortex evolution. Trends Neurosci. *43*, 843–853.

Kim, J.H., Lee, S.R., Li, L.H., Park, H.J., Park, J.H., Lee, K.Y., Kim, M.K., Shin, B.A., and Choi, S.Y. (2011). High cleavage efficiency of a 2A peptide derived from porcine teschovirus-1 in human cell lines, zebrafish and mice. PLoS One *6*, e18556.

Kobayashi, T., and Kageyama, R. (2011). Hes1 oscillations contribute to heterogeneous differentiation responses in embryonic stem cells. Genes *2*, 219–228.

Kobayashi, T., Mizuno, H., Imayoshi, I., Furusawa, C., Shirahige, K., and Kageyama, R. (2009). The cyclic gene Hes1 contributes to diverse differentiation responses of embryonic stem cells. Genes Dev. *23*, 1870–1875.

Li, S., Xue, H., Wu, J., Rao, M.S., Kim, D.H., Deng, W., and Liu, Y. (2015). Human induced pluripotent stem cell NEUROG2 dual knockin reporter lines generated by the CRISPR/Cas9 system. Stem Cells Dev. *24*, 2925–2942.

Meyer, G., and González-Gómez, M. (2018). The heterogeneity of human Cajal-Retzius neurons. Semin. Cell Dev. Biol. *76*, 101–111.

Meyer, G., Schaaps, J.P., Moreau, L., and Goffinet, A.M. (2000). Embryonic and early fetal development of the human neocortex. J. Neurosci. *20*, 1858–1868.

Nagashima, F., Suzuki, I.K., Shitamukai, A., Sakaguchi, H., Iwashita, M., Kobayashi, T., Tone, S., Toida, K., Vanderhaeghen, P., and Kosodo, Y. (2014). Novel and robust transplantation reveals the acquisition of polarized processes by cortical cells derived

from mouse and human pluripotent stem cells. Stem Cells Dev. *23*, 2129–2142.

Santana, J., and Marzolo, M.P. (2017). The functions of Reelin in membrane trafficking and cytoskeletal dynamics: implications for neuronal migration, polarization and differentiation. Biochem. J. *474*, 3137–3165.

Tabata, H., and Nakajima, K. (2003). Multipolar migration: the third mode of radial neuronal migration in the developing cerebral cortex. J. Neurosci. *23*, 9996–10001.

Wu, J., Matthias, N., Lo, J., Ortiz-Vitali, J.L., Shieh, A.W., Wang, S.H., and Darabi, R. (2018). A myogenic double-reporter human pluripotent stem cell line allows prospective isolation of skeletal muscle progenitors. Cell Rep. *25*, 1966–1981.e4.

Zhang, J.Z., Termglinchan, V., Shao, N.Y., Itzhaki, I., Liu, C., Ma, N., Tian, L., Wang, V.Y., Chang, A.C.Y., Guo, H., et al. (2019). A human iPSC double-reporter system enables purification of cardiac lineage subpopulations with distinct function and drug response profiles. Cell Stem Cell *24*, 802–811.e5.

Stem Cell Reports, Volume *17*

Supplemental Information

Direct visualization of the transition status during neural differentiation by dual-fluorescent reporter human pluripotent stem cells

Gwanghyun Park, Minkyung Shin, Wonyoung Lee, Akitsu Hotta, Taeko Kobayashi, and Yoichi Kosodo

Supplemental information

Supplemental Figures

A

RGEN target for TUBB3	Chromosome	Position	Direction	GC contents (%)	Mismatches			Target name
					0	1	2	
CGAGATGTACGAAGACGACGAGG	Chr16	89935752	+	55.0	1	0	0	G2
GTACATCTCGCCCTCTTCCTCGG	Chr16	89935739	-	55.0	1	0	0	G3
GATGTACGAAGACGACGAGGAGG	Chr16	89935523	-	65.0	1	0	1	G4
**RGEN target for NGN2**	**Chromosome**	**Position**	**Direction**	**GC Contents (%)**	**Mismatches**			**Target name**
					**0**	**1**	**2**	
CCGGGTCAGACATGGACTATTGG	Chr4	112514722	-	55.0	1	0	0	T1
CACTTTATCGCCCGCCAGCCCGG	Chr4	112514745	-	65.0	1	0	0	T2
TAGATACAATCCCTGGCTATGGG	Chr4	112514657	+	40.0	1	0	0	T3
TGGGGAGGTGAGGTGCATAGCGG	Chr4	112514676	+	60.0	1	0	0	T4
GCAGCTCTAGATACAATCCCTGG	Chr4	112514650	-	50.0	1	0	2	T5

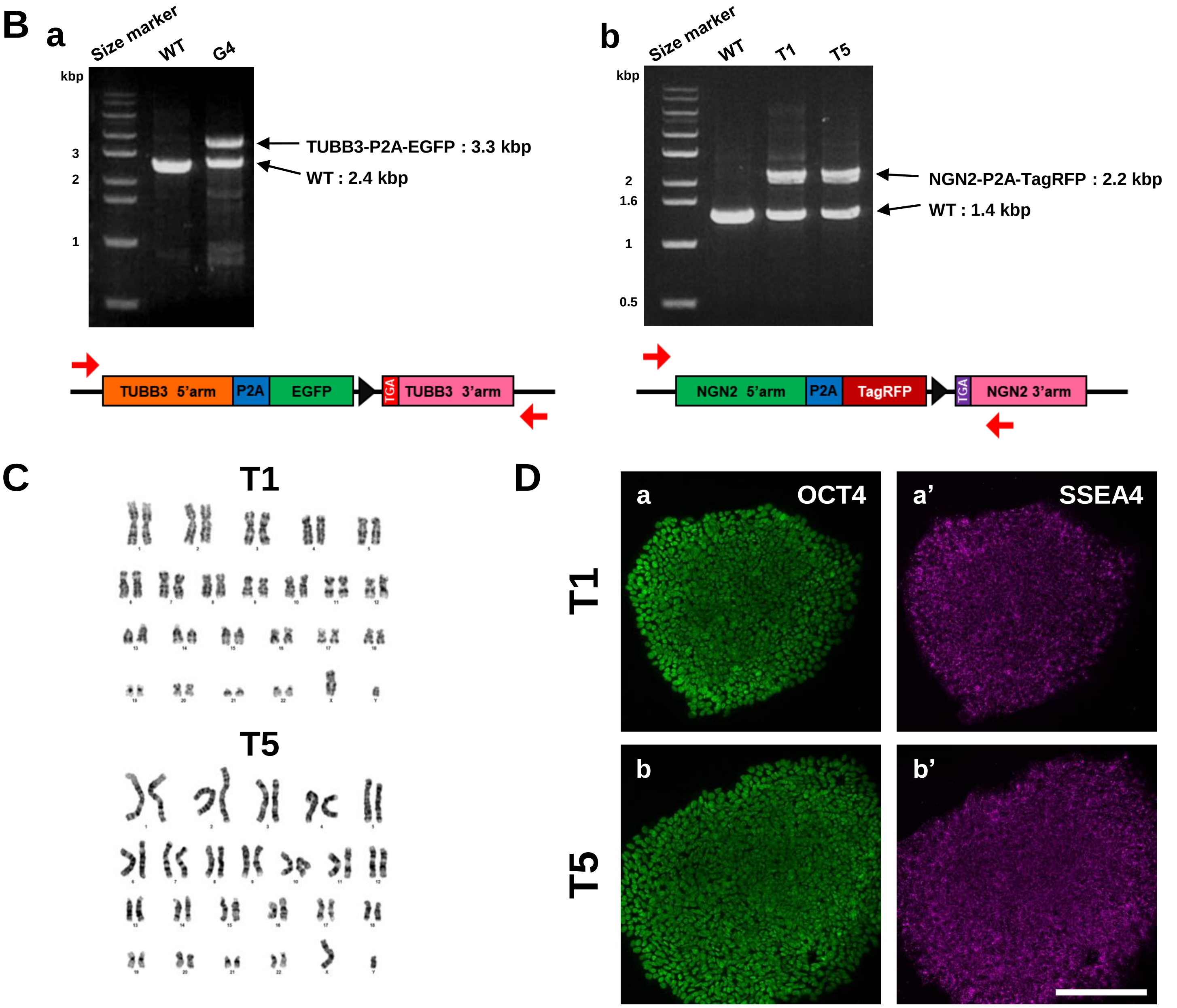

Supplemental Figure 1. Construction of TUBB3^{EGFP} and NGN2^{TagRFP} dual-reporter hiPSCs, Related to Figure 1A

A. Sequences of the sgRNAs used to construct TUBB3^{EGFP} and NGN2^{TagRFP} .

B. Confirmation of heterozygous KI by PCR. The arrows indicate the positions of the primers (a, TUBB3-CRISPR-confirm-F1 and TUBB3-CRISPR-confirm-R2; b, NGN2-genome-F1 and NGN2-CRISPR-confirm-R3, each primer sequence is indicated in Table S1).

C. Karyotype analysis of dual-reporter hiPSCs (T1 and T5)

D. Immunostaining images obtained using antibodies for OCT4 (green) for T1 (a) and T5 (b) and SSEA4 (magenta) for T1 (a') and T5 (b') fixed at the pluripotent stage. Bar = 200 µm.

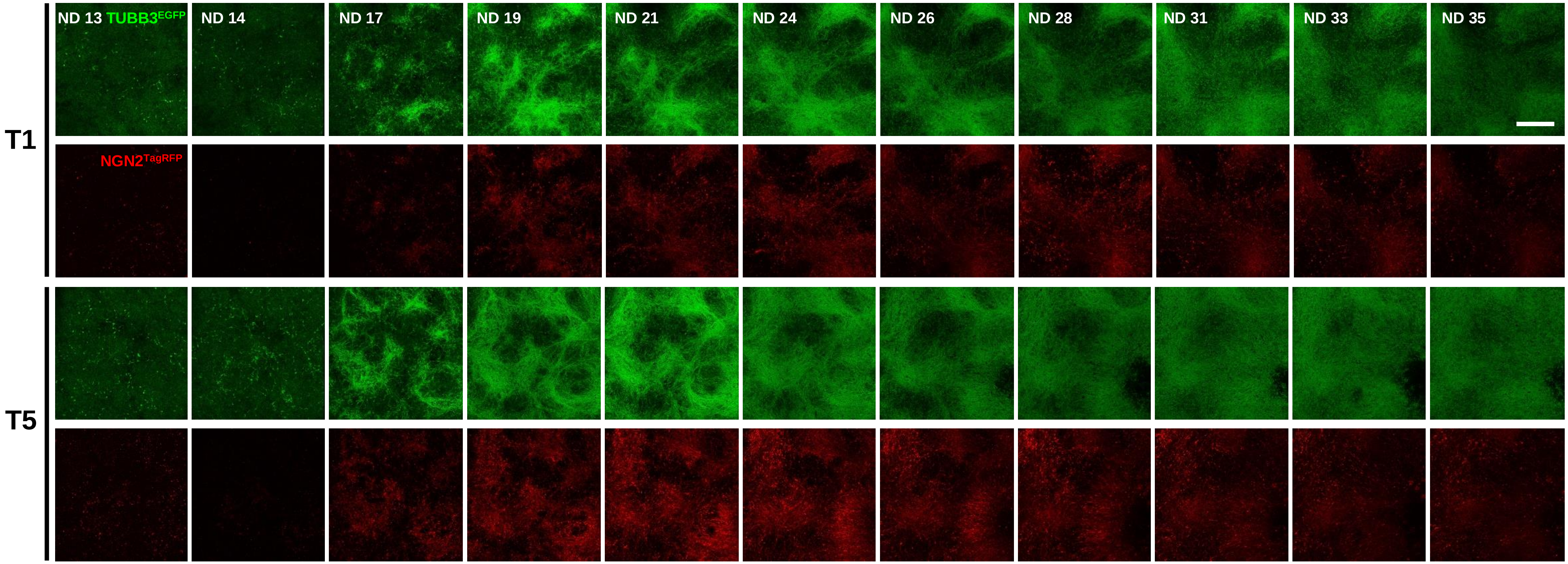

Supplemental Figure 2. Sequential images of TUBB3^{EGFP} and NGN2^{TagRFP} expression in dual-reporter cells, Related to Figure 1C
Sequential images of TUBB3^{EGFP} and NGN2^{TagRFP} expression in dual-reporter cells (T1 and T5) during neural differentiation (from ND 13 to 35) at the same position on the grid culture dish. Bar = 300 µm.

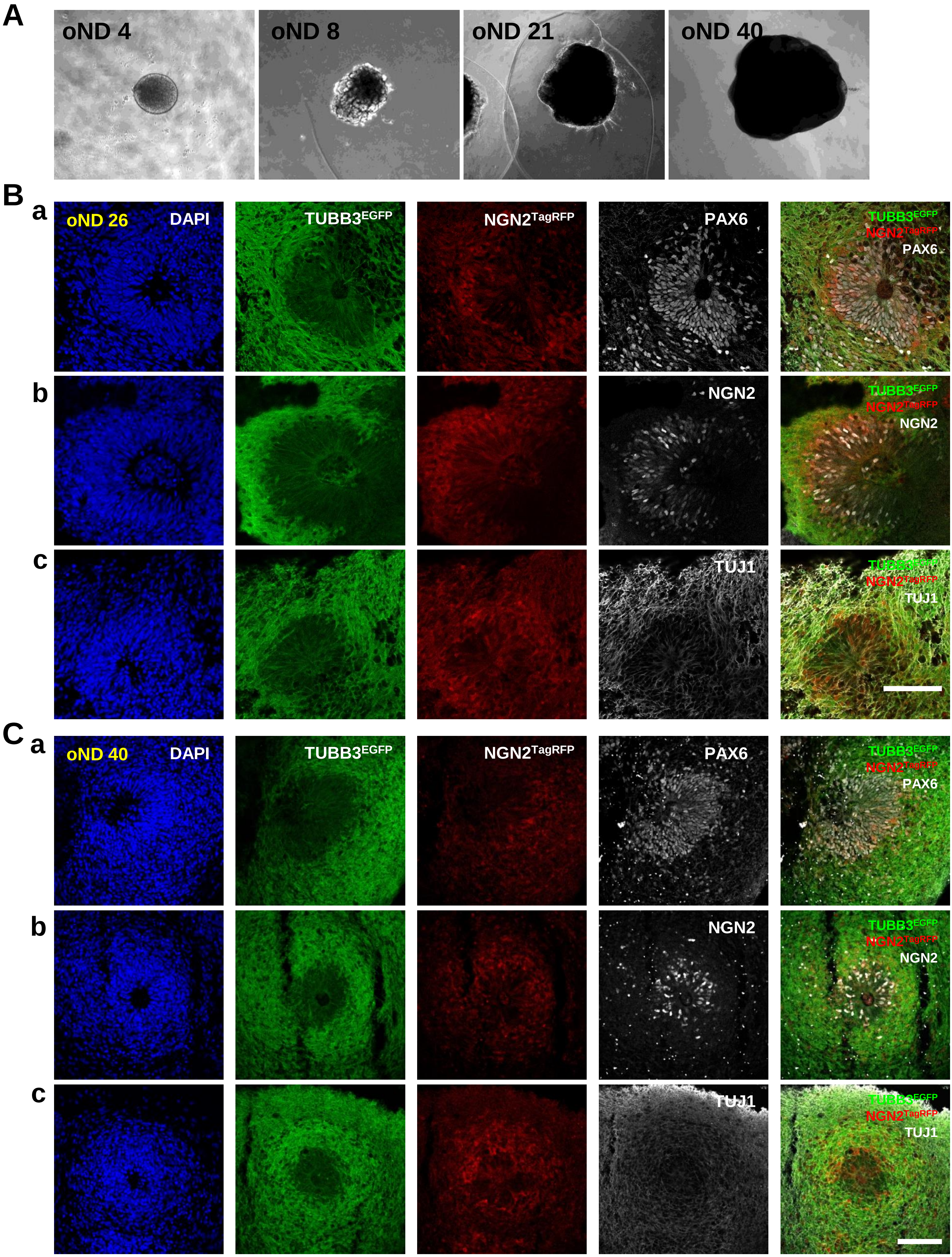

Supplemental Figure 3. Cerebral organoids generated from dual-reporter hiPSCs, Related to Figure 2A

A. Complete views of cerebral organoids on oND 4, 8, 21, and 40.

B. Immunostaining images of organoids obtained using antibodies against PAX6 (a), NGN2 (b), and TUJ1 (c) on oND 26.

C. Immunostaining images of organoids obtained using antibodies against PAX6 (a), NGN2 (b), and TUJ1 (c) on oND 40.

Bars = 100 µm.

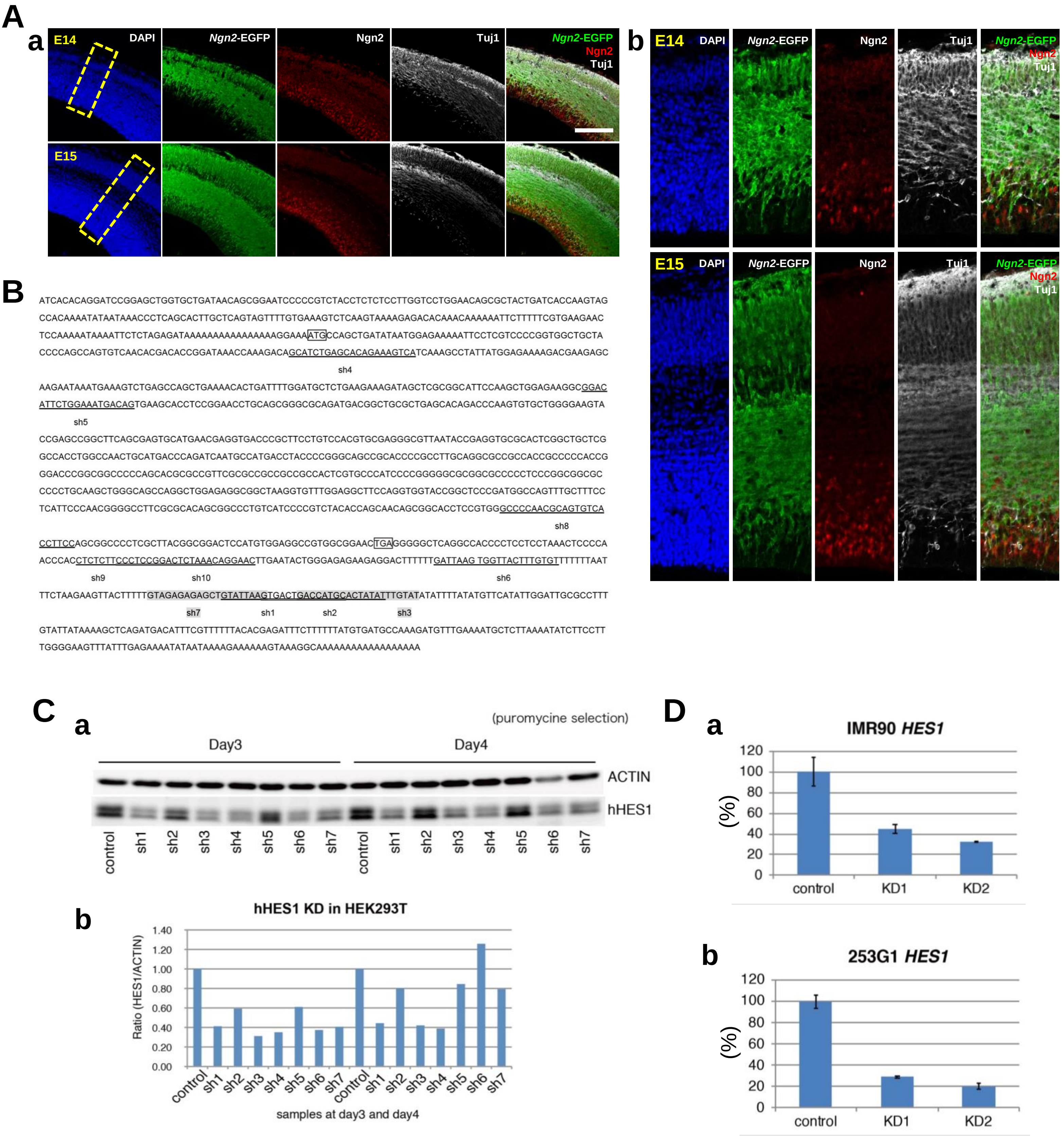

Supplemental Figure 4. Expression of *Ngn2*-EGFP in the developing mouse brain, Related to Figure 2A and confirmation of HES1 KD in HEK293T cells and other hiPSC lines, Related to Figure 3D

A. Immunostaining images of embryonic brain sections of *Ngn2*-EGFP mice obtained using antibodies against Ngn2 (red) and Tuj1 (white) at E14.5 and E15.5. Bar = 200 μm.

B. Candidate shRNA sequences for HES1 KD. Note that sh3 = KD1 and sh4 = KD2.

C. Western blotting to quantify the endogenous HES1 expression in HEK293T cells at 3 and 4 days after the transfection of KD constructs.

D. qRT–PCR analyses of HES1 in IMR90 (a) and 253G1 (b) hiPSC lines after the transfection of two different shRNA constructs (KD1 and KD2) at the pluripotent stage. The expression level was normalized to that in the control.

Supplemental Movie Legends

Movie S1
Time-lapse movie file for Figure 1D (12 min/frame)

Movie S2
Time-lapse movie file for Figure 4A (12 min/frame)

Table S1. Primer and antibody list

Primers for qRT-PCR		
**Gene**	**Forward primer**	**Reverse primer**
GAPDH	CGCTCTCTGCTCCTCCTGTT	CCATGGTGTCTGAGCGATGT
HES1	CGGACATTCTGGAAATGACA	TACTTCCCCAGCACACTTGG
NGN2	ACCATCTCACTTCACAGGGCAG	TCAAGAAGCGCCCCCAAAAC
TBR2	CACCGCCACCAAACTGAGAT	CGAACACATTGTAGTGGGCAG
TBR1	ATGGGCAGATGGTGGTTTTA	GACGGCGATGAACTGAGTCT
TUBB3	CCTGGAACCCGGAACCAT	AGGCCTGAAGAGATGTCCAAAG
Albumin	TTTGCAGATGTCAGTGAAAGAGA	TGGGGAGGCTATAGAAAATAAGG
WPRE	GTCCTTTCCATGGCTGCTC	CCGAAGGGACGTAGCAGA

Primers for knock-in generation and confirmation		
**Primer**	**Sequence**	**Experiment**
TUBB3-5'-fwd-1	AAAGCTGGGTCTAGAGTCAGAGTGGGGCCGGCA	TUBB3-EGFP knock-in donor DNA cloning
G4-rev-1	AGTAGCTCCGCTTCCCTTGGGGCCCTGGGCCTCCGACTCTTC	
2A-EGFP-fwd-1	GCCCAGGGCCCCAAGGGAAGCGGAGCTACTAACT	
EGFP-rev-1	TTTAAACGCGGCCGCTTACTTGTACAGCTCGTC	
TUBB3-3'-fwd-1	CGAAGTTATGAATTCTGAAGCTGCTCGCAGCTGGA	
TUBB3-3'-rev-1	ACCAATTCAGTCGACCGGCCTCTCTGGGCTCCA	
sgRNA-G4-fwd	GAGACCACTTGGATCCGATGTACGAAGACGACGAGGGTTTTAGAGCTAGAAATAGCA	TUBB3-EGFP knock-in guide RNA cloning
sgRNA-Universal-rev	GCCCGGGTTTGAATTCAAAAAAAGCACCGACTCGGTGCCACTTTTTCAAGTTGATAACGGACTAGCCTTATTTTAACTTGCTATTTCTAGCTC	
TUBB3-CRISPR-confirm-F1	GTCTGGACTGCAGAGTCCCTGG	TUBB3-EGFP knock-in genotyping and sequencing
EGFP-reverse-R1	GACACGCTGAACTTGTGGCCG	
Puro-confirm-F2	GCAACCTCCCCTTCTACGAG	
TUBB3-CRISPR-confirm-R2	GCAGAAGGATCCAGCTAACACGCTG	
TUBB3-CRISPR- confirm-F3	TTGCGTGTGGCAGTTTTAAG	
TUBB3-CRISPR- confirm-R3	AAGGACATCTCTGGCACCAA	
NGN2-5'arm-F-1	AAAGCTGGGTCTAGAGATGTTCGTCAAATCCGAGA	NGN2-TagRFP knock-in donor DNA cloning
NGN2-5'arm-R	AGTAGCTCCGCTTCCGATACAATCCCTGGCTATGGGGAGGTGAGGTGCATAGCGGTGCTTGTCGGGAGGTGGGGGCTGCCAATAGTCCATGTCCGATCCCGCCGGGCTAGCGGGTGA TAAGGTGCA	
2A-RFP-F	GCCAGGGATTGTATCGGAAGCGGAGCTACTAACTTCAGCCTGCTGAAGCAGGCTGGAGACGTGGAGGAGAACCCTGGACCTATGGTGTCTAAGGGCGAAG	
2A-RFP-R-1	TTTAAACGCGGCCGCTCAATTAAGTTTGTGCCCCA	
NGN2 3'arm-F	CGAAGTTATGAATTCTAGAGCTGCCATTTCTGCTA	
NGN2 3'arm-R	CAGTCGACTGGATCCTGCTTAGGTTTTCAGACTGT	
sgRNA-Target1-fwd	GAGACCACTTGGATCCCCGGGTCAGACATGGACTATGTTTTAGAGCTAGAAATAGCA	NGN2-TagRFP knock-in guide RNA cloning
sgRNA-Target5-fwd	GAGACCACTTGGATCCGCAGCTCTAGATACAATCCCGTTTTAGAGCTAGAAATAGCA	
NGN2-CRISPR-confirm-fwd-1	GCAGCTGGATGGCAAATAAT	NGN2-TagRFP knock-in genotyping and sequencing
NGN2-CRISPR-confirm-rev-1	TCAGCTCTTCGCCCTTAGAC	
NGN2-CRISPR-confirm-fwd-2	GCAACCTCCCCTTCTACGAG	
NGN2-CRISPR-confirm-rev-2	CTGATAGTGACCTGTTCGTTGC	
NGN2-CRISPR-confirm-F3	AATTCCACCTCCCCCTACAG	
NGN2-CRISPR-confirm-R3	GCCCGTCTGAATGAAGGATA	
hEF1a-F1	GACGGGGGTAGTCTCAAGC	
hEF1a-R1	CTTTCCGCTCACGCAACT	
NGN2-genome-F1	AGCCACCGCTTCCAACTAC	

Antibodies							
**Antibody**	**Company**	**Catalog number**	**Dilution**	**Antibody**	**Company**	**Catalog number**	**Dilution**
GFP	Aves	GFP-1010	1:1000	BrdU	Abcam	ab6326 (BU1/75)	1:1000
RFP	Evrogen	AB233	1:1000	anti-chicken Alexa488	Jackson Immuno Research	703-545-155	1:1000
TUJ1	Biolegend	MMS-435P	1:1000	anti-rabbit Alexa555	Invitrogen	A31572	1:1000
NGN2	CST	13144	1:200	anti-mouse Alexa647	Invitrogen	A31571	1:1000
KI67	BD	556003	1:1000	anti-rabbit Alexa647	Invitrogen	A31573	1:1000
PAX6	MBL	PD022	1:1000	anti-rabbit Alexa647	Invitrogen	A21245	1:1000
TBR1	Abcam	ab31490	1:1000	anti-rat Alexa647	Invitrogen	A21247	1:1000
CTIP2	Abcam	ab18465	1:1000	anti-rabbit Alexa405	Invitrogen	A48258	1:1000
SATB2	Abcam	ab51502	1:1000	anti-goat Alexa647	Invitrogen	A21447	1:1000
RELN	R&D	AF3820	1:100				

Supplemental experimental procedures

Animals

Pregnant ICR mice were purchased from Core Tech, South Korea. *Neurog2*-EGFP transgenic mice were obtained from the GENSAT project (#000266-UNC) and bred at KBRI by crossing with C57BL/6 mice. The day of birth was defined as postnatal day 0 (P0).

Neural differentiation

The expansion and neural differentiation of hiPSCs were performed using a previously described method (Espuny-Camacho et al., 2013) with some modifications. HiPSCs were maintained in StemFit Basic 04 medium (Ajinomoto, AJ200B, Japan) in a multiwell dish (Corning, USA) coated with Vitronectin XF (Stemcell Technologies, 07180, Canada). For neural differentiation, cells were plated on Vitronectin XF-coated dishes at a defined density (6.0 x 10^4 cells/well in a 6-well plate) in StemFit Basic 04 medium containing Y-27632 (ROCK inhibitor; 10 μM) (Wako, 036-24023, Japan) two days prior to neural differentiation. On the day of differentiation initiation (defined as "ND 0"), the medium was replaced with neural differentiation medium supplemented with N2 (R&D Systems, AR009, USA), B27 (Gibco, 17054-044, USA) and Noggin (for ND 0-16, 100 ng/ml, R&D Systems, 6057-NG-100, USA) and changed every 2 days. On ND 4 or 5, cells were replated using TrypLE Select CTS (Gibco, A12859-01, USA) diluted in 0.5 mM EDTA (Invitrogen, 15575-038, USA) at a density of 2.0 x 10^5 cells/well in a 6-well plate. The medium was changed every 2 days and included Y-27632 for 2 days after replate and noggin until ND 16. In the DAPT treatment assay, DAPT was added to the medium beginning on ND 11 at a concentration of 5 μM. To evaluate gene expression in hiPSCs, qRT–PCR was performed as described previously (Iwashita et al., 2019) using specific primer sets (Table S1).

Design and cloning of the KI constructs

CRISPR RGEN Tools (Bae et al., 2014) was used to find candidates for single-guide RNA (sgRNA) target sites near the stop codon of the NGN2 or TUBB3 coding sequence and to evaluate off-target scores. Three and five target sites were selected for the $TUBB3^{EGFP}$ and $NGN2^{TagRFP}$ KI constructs, respectively (Figure 1A and S1). To clone a DNA-coding sgRNA sequence into an expression vector, a pair of oligonucleotides (a forward primer containing the sgRNA target sequence and a reverse primer containing an sgRNA scaffold sgRNA) was designed according to a previously described strategy (Li et al., 2016). After PCR amplification using the primer set, the PCR fragment was cloned into pHL-H1-ccdB-mEF1α-RiH (Addgene ID: 60601). To construct donor DNA, P2A-TagRFP, P2A-EGFP, 5' and 3' homology arms of NGN2 and TUBB3 genes were amplified by PCR using genomic DNA extracted from wild type hiPSCs, then ligated by In-Fusion system (Takara, 639648 Japan) into pENTR-DMD-Donor vector (Addgene ID:60605), which has floxed EF1a-Puro cassette. P2A-TagRFP and P2A-EGFP cassettes were inserted just before the stop codon of the coding sequence of NGN2 or TUBB3 genes. The primers and vectors used in this study are listed in Table S1.

Electroporation

To establish dual-reporter cell lines, DNA constructs were sequentially electroporated (EP) into hiPSCs (Figure 1A). The first round of EP was carried out to establish $TUBB3^{EGFP}$ KI cells. A Cas9 expression plasmid (Addgene ID: 60599) (Li et al., 2016), an sgRNA expression and donor plasmids were cotransfected into 1.0 x 10^6 hiPSCs using a NEPA21 electroporator (Nepagene, Japan) under previously described conditions (voltage of 125 V and electroporation pulse width of 5 ms (Oceguera-Yanez et al., 2016)). Immediately after EP, the cells were transferred into medium containing Y-27632 to prevent apoptosis. To select cells carrying the donor DNA sequence, puromycin (Sigma, P9620, USA, 250 ng/ml in medium) was administered 2 to 5 days after EP. After the passaging step, low-cell density culture was performed (300 cells in a well in a 6-well dish) until small colonies were formed, and single colonies were then selected and transferred to the wells of 24-well dishes. After expansion, KI candidate clones were selected by genotyping (Figure S1) using the primer sets (Table S1). Subsequently, the second round of EP was performed with CAG-Cre and CAG-RFP (Shitamukai et al., 2011) to remove the floxed-

puromycin cassette by Cre recombination. Low-cell density culture followed by genotyping by the primer sets (Table S1) allowed the selection of RFP-positive clones excised the puromycin cassette by the Cre plasmid function. After the expansion step, the third round of EP was performed with the sgRNA and the donor DNA sequence to generate $NGN2^{TagRFP}$ KI cells using the method described for the first EP. Two independent clones (T1 and T5) were selected after the neural induction of 4 clones each for T1 and T5, followed by the fourth round of EP to remove the puromycin cassette by the Cre plasmid. All clones were heterozygous KI cells (Figure S1), and successful genome editing was confirmed by sequencing the corresponding genomic loci using specific primer sets (Table S1). Confirmation of karyotypes (Figure S1) was conducted by Samkwang Medical Laboratory (Republic of Korea).

Cerebral organoid generation

Cerebral organoids were generated using a STEMdiff Cerebral Organoid Kit (Stemcell Technology Cat# 08570, Canada) according to the manufacturer's instructions. Briefly, hiPSCs maintained in StemFit Basic 04 were detached by TrypLE Select CTS diluted in 0.5 mM EDTA and transferred to an ultralow attachment 96-well plate (Corning, 7007, USA) at a density of 9.0 x 10^3/well in embryoid body (EB) formation medium containing Y-27632 (10 μM). For mixed organoid generation, 8.1 x 10^3 original hiPSCs and 9.0 x 10^2 $TUBB3^{EGFP}$/$NGN2^{TagRFP}$ reporter hiPSCs were mixed and transferred into each well. The medium was changed every 2 days without adding Y-27632. To induce cerebral organoid formation, 1 or 2 EBs were moved to a 24-well ultralow attachment plate (Corning, 3473, USA) containing induction medium. After 7 days, organoids were-embedded in Matrigel (Corning, 354230, USA) and cultured with Expansion medium. After 10 days, the organoids were matured on an orbital shaker inside a CO_2 incubator and cultured for up to 40 days. BrdU (Sigma, B9285, USA, 1 μM in medium) was added on oND 26 cells for 4 h before fixation. For double labeling, EdU (Invitrogen, C10340, USA, 4 μM) was added to the cells for 4 h on oND 26, and the cells were cultured after washing with medium. After 2 weeks, BrdU was added on oND 40 for 4 h prior to fixation.

Transplantation

Transplantation of hiPSC-derived neural cells to the developing mouse brain was performed using the highly efficient transplantation method published previously (Nagashima et al., 2014) with slight modifications. Briefly, neural differentiation from dual-reporter hiPSCs was conducted using the 2D culture method. On ND 20 and 27, cells were detached from the culture dish using Accutase (Gibco, A1110501, USA) and used to make a 50 μl mixed solution containing (1.0x10^5 cells/μl) donor cells, 20 mM ethylene glycol tetraacetic acid (EGTA), 10 μM Y-27632, and 0.01% Fastgreen. One to two microliters of the mixed solution was injected into the lateral ventricles of E14.5 ICR mice *in utero*, and neonatal brains were then dissected and fixed with 4% paraformaldehyde (PFA) at P0. To observe the donor cells in the host brain tissue, 100-μm vibratome sections were prepared and subjected to immunostaining analysis using antibodies against GFP (Aves, GFP-1010, USA) and RFP (Evrogen, AB233, Russia) to enhance the signals of $TUBB3^{EGFP}$ and $NGN2^{TagRFP}$, respectively. Before observing the stained vibratome sections, CUBIC treatment was performed for tissue clearing. CUBIC was prepared according to a previous study (Susaki et al., 2014), and stained tissue sections were immersed in 100 μl for 5-10 min with gentle shaking. Fluorescence 3D images were acquired by confocal microscopy (Andor Technology, Dragonfly 502w, UK) using 488 and 561 nm lasers. Z-stack sections were acquired at 0.5 μm intervals between each optical section using a 40x objective lens.

KD of HES1 function

To make the DNA construct, the following oligos containing the target sequence for human HES1 were inserted into the BbsI site of the psiRNA-h7SKneo plasmid (InvivoGen, USA): ACCTC-GGGTTGTTACtTAAGTCCTgGA-TCAAGAG-TCTAGGACTTAGGTAACATCC-TT (control scramble), ACCTC-GAtCATGCgCTgTATTTGTAT-TCAAGAG-ATACAAATATAGTGCATGGTC-TT (HES1-sh3 for KD1), and ACCTC-GCgTCTGgGCgCAGAAAGTCA-TCAAGAG-TGACTTTCTGTGCTCAGATGC-TT (HES1-sh4 for KD2). The ACCTC on the 5' side and the TT on the 3' side were added to generate the restriction enzyme site, and the TCAAGAG sequence at the center was added to generate the loop for the short hairpin. To prevent mutation during replication, 3 point mutations (indicated by lowercase letters) were inserted. The sequence

containing both the h7SK promoter and inserts (h7SK-shRNA) was then transferred to the CSII vector containing PGK-neo-pA. All h7SK-shRNAs were inserted into PGK-neo-pA in CSII at the 3' side. To produce lentivirus for HES1 KD, HEK293T cells were plated in DMEM/F12 supplemented with 10% FBS and 1% penicillin/streptomycin (3.0 x 10^6 cells on a 10 cm dish) 24 h before transfection. HEK293T cells were cotransfected with a CSII vector containing the KD constructs (8.4 µg), psPAX2 (6.2 µg) and pMD2. G (2.6 µg) using PEI MAX (Polysciences, 24765, USA) for lentivirus packaging. Media containing forskolin (Wako, 067-02191, Japan, 10 µM) were changed at 8 h after transfection. Media containing lentivirus were collected at 54 h after transfection, passed through a 0.45 µm filter and concentrated by centrifugation. For titration, 5.0 x 10^4 HEK293T cells were infected with serial dilutions of lentivirus in a well of a 12-well plate. Genomic DNA was extracted 4 days after infection, and the titer was measured by qRT–PCR (Barczak et al., 2015). TUBB3^{EGFP}/NGN2^{TagRFP} reporter hiPSCs were infected with lentivirus at an MOI of 1 after dissociation and selected with 250 µg/ml G418 (Roche, 4727878001, Switzerland) for 24 h after 4-6 days.

Immunostaining
Cells cultured on cover glass were fixed with 4% PFA for 10 min. Fixed samples were permeabilized with 0.5 x Triton X-100 in PBS for 10 min and then incubated in blocking solution (2% BSA in PBS) for 1 h at room temperature. Subsequently, samples were incubated with primary antibodies diluted in the blocking solution for 2 h at room temperature or overnight at 4°C. After rinsing with PBS, fluorochrome-conjugated secondary antibodies and DAPI diluted in PBS were applied for 1 h at room temperature. Samples were mounted with a drop of mounting medium (Invitrogen, P36961, USA) and observed by confocal microscopy (Leica, SP8, Germany). Organoids were fixed with 4% PFA overnight at 4°C and then with 20% sucrose solution overnight at 4°C. Organoids were initially embedded in gelatin/sucrose solution (Lancaster and Knoblich, 2014) and then re-embedded in OCT compound (SAKURA, 4583, Japan) to make a frozen block. The tissue blocks were sectioned at a thickness of 20 µm for immunostaining. Information regarding the antibodies used in this study is provided in Table S1.

Live cell imaging
To record the transient expression of fluorescent reporters during neural differentiation for several weeks, snapshot imaging of EGFP and TagRFP was performed on cells cultured on a glass bottom dish with grid lines (IWAKI, 3922-035, Japan). Using the grid line as a landmark, it was possible to find cells in the same position under the bright field of the confocal microscope on consecutive days. For live imaging of cell division, a glass bottom dish without grid lines (Nunc, 150682, USA) was used. To maintain the cell survival environment during microscope imaging, the incubation chamber (LCI, Stage-top Incubator System TC, Korea) was set at 5% CO_2 and 37°C. Cells were maintained in a conventional CO_2 incubator when not being imaged. To track the cell division and migration of cells expressing fluorescent reporters, time-lapse imaging was performed with a confocal microscope (Leica, SP8, Germany) for 12-15 h at 10-15 min intervals in the incubation chamber.

Image analysis
The fluorescence intensities of EGFP and TagRFP were quantified using ImageJ software (https://imagej.nih.gov/ij/download.html). The background was removed by the subtract background tool, and the rolling ball radius parameter was set to at least the size of the cell that was not part of the background. The threshold was adjusted to distinguish the components of interest and the background. The intensity was measured automatically based on the threshold images. Sholl analysis of differentiating cells was performed using the Simple Neurite Tracer (SNT) function in ImageJ as previously described (Binley et al., 2014). Sholl analysis was performed using the tracing images, and concentric rings spaced 3.5 µm apart centered on the soma were applied.

Cell migration analysis
Time-lapse recordings of cell migration during neural differentiation were analyzed using Imaris software (Oxford Instruments, x64 9.0.2, UK). Cells were marked as spots using the Spot tool, and the migration of each cell was tracked over time automatically. For the filter type, “quality for

classify spots" was selected, and the lower threshold parameter was set to 8.00. The autoregressive motion algorithm was selected using 20 μm for the MaxDistance and 3 μm for the MaxGapSize settings. Statistical values of track straightness (TS) and track speed max (TSM) were utilized to characterize the cellular migration pattern.

Cell division analysis

Fluorescent signal intensity of time-lapse recorded images including cell division were analyzed using ImageJ software. G^+/R^- neurons identified in the analyzed frame were considered mature neurons. The fluorescent signal was divided by the area calculated by outlining cell bodies of mature neurons or dividing cells. The EGFP signal of G^+/R^- neurons was set as the normalization standard (100%) to calculate relative intensity for dividing cells.

Supplemental Information References

Bae, S., Park, J., and Kim, J.S. (2014). Cas-OFFinder: a fast and versatile algorithm that searches for potential off-target sites of Cas9 RNA-guided endonucleases. Bioinformatics *30*, 1473-1475.

Barczak, W., Suchorska, W., Rubis, B., and Kulcenty, K. (2015). Universal real-time PCR-based assay for lentiviral titration. Mol Biotechnol *57*, 195-200.

Binley, K.E., Ng, W.S., Tribble, J.R., Song, B., and Morgan, J.E. (2014). Sholl analysis: a quantitative comparison of semi-automated methods. J Neurosci Methods *225*, 65-70.

Espuny-Camacho, I., Michelsen, K.A., Gall, D., Linaro, D., Hasche, A., Bonnefont, J., Bali, C., Orduz, D., Bilheu, A., Herpoel, A.*, et al.* (2013). Pyramidal neurons derived from human pluripotent stem cells integrate efficiently into mouse brain circuits in vivo. Neuron *77*, 440-456.

Iwashita, M., Ohta, H., Fujisawa, T., Cho, M., Ikeya, M., Kidoaki, S., and Kosodo, Y. (2019). Brain-stiffness-mimicking tilapia collagen gel promotes the induction of dorsal cortical neurons from human pluripotent stem cells. Sci Rep *9*, 3068.

Lancaster, M.A., and Knoblich, J.A. (2014). Generation of cerebral organoids from human pluripotent stem cells. Nat Protoc *9*, 2329-2340.

Li, H.L., Gee, P., Ishida, K., and Hotta, A. (2016). Efficient genomic correction methods in human iPS cells using CRISPR-Cas9 system. Methods *101*, 27-35.

Nagashima, F., Suzuki, I.K., Shitamukai, A., Sakaguchi, H., Iwashita, M., Kobayashi, T., Tone, S., Toida, K., Vanderhaeghen, P., and Kosodo, Y. (2014). Novel and robust transplantation reveals the acquisition of polarized processes by cortical cells derived from mouse and human pluripotent stem cells. Stem Cells Dev *23*, 2129-2142.

Oceguera-Yanez, F., Kim, S.I., Matsumoto, T., Tan, G.W., Xiang, L., Hatani, T., Kondo, T., Ikeya, M., Yoshida, Y., Inoue, H.*, et al.* (2016). Engineering the AAVS1 locus for consistent and scalable transgene expression in human iPSCs and their differentiated derivatives. Methods *101*, 43-55.

Shitamukai, A., Konno, D., and Matsuzaki, F. (2011). Oblique radial glial divisions in the developing mouse neocortex induce self-renewing progenitors outside the germinal zone that resemble primate outer subventricular zone progenitors. J Neurosci *31*, 3683-3695.

Susaki, E.A., Tainaka, K., Perrin, D., Kishino, F., Tawara, T., Watanabe, T.M., Yokoyama, C., Onoe, H., Eguchi, M., Yamaguchi, S.*, et al.* (2014). Whole-brain imaging with single-cell resolution using chemical cocktails and computational analysis. Cell *157*, 726-739.
